# LncRNA TTN-AS1 promotes the progression of cholangiocarcinoma via the miR-320a/neuropilin-1 axis

**DOI:** 10.1038/s41419-020-02896-x

**Published:** 2020-08-15

**Authors:** Huaqiang Zhu, Bo Zhai, Changjun He, Ziyi Li, Hengjun Gao, Zheyu Niu, Xian Jiang, Jun Lu, Xueying Sun

**Affiliations:** 1 Department of Hepatobiliary Surgery, Shandong Provincial Hospital Affiliated to Shandong First Medical University, 250021 Jinan, China; 2 The Hepatosplenic Surgery Center, the First Affiliated Hospital of Harbin Medical University, 150001 Harbin, China; 3 Department of General Surgery, the Fourth Affiliated Hospital of Harbin Medical University, 150001 Harbin, China; 4 Department of Surgery, the Third Affiliated Hospital of Harbin Medical University, 150081 Harbin, China

**Keywords:** Bile duct cancer, Long non-coding RNAs, miRNAs, Bile duct cancer

## Abstract

Neuropilin-1 regulated by miR-320a participates in the progression of cholangiocarcinoma by serving as a co-receptor that activates multiple signaling pathways. The present study sought to investigate upstream lncRNAs that control the expression of miR-320a/neuropilin-1 axis and dissect some of the underlying mechanisms. Here we report lncRNA TTN-AS1 (titin-antisense RNA1) acts as a sponging ceRNA to downregulate miR-320a and is highly expressed in human cholangiocarcinoma tissues and cells. The expression of the above three molecules is correlated with the clinicopathologic parameters of cholangiocarcinoma patients. In this study, multiple bioinformatics tools and databases were employed to seek potential lncRNAs that have binding sites with miR-320a and TTN-AS1 was identified because it exhibited the largest folds of alteration between cholangiocarcinoma and normal bile duct epithelial cells. The regulatory role of TTN-AS1 on miR-320a was further evaluated by luciferase reporter and RNA pulldown assays, coupled with in situ hybridization and RNA immunoprecipitation analyses, which showed that TTN-AS1 bound to miR-320a through an argonaute2-dependent RNA interference pathway in the cytoplasm of cholangiocarcinoma cells. Knockdown and overexpression assays showed that the regulatory effect between TTN-AS1 and miR-320 was in a one-way manner. TTN-AS1 promoted the proliferation and migration of cholangiocarcinoma cells via the miR-320a/ neuropilin-1 axis. The function of TTN-AS1 on tumor growth and its interaction with miR-320a were confirmed in animal models. Further mechanistic studies revealed that TTA-AS1, through downregulating miR-320a, promoted cell cycle progression, epithelial–mesenchymal transition, and tumor angiogenesis by upregulating neuropilin-1, which co-interacted with the hepatocyte growth factor/c-Met and transforming growth factor (TGF)-β/TGF-β receptor I pathways. In conclusion, the present results demonstrate that lncRNA TTA-AS1 is a sponging ceRNA for miR-320a, which in turn downregulates neuropilin-1 in cholangiocarcinoma cells, indicating these three molecules represent potential biomarkers and therapeutic targets in the management of cholangiocarcinoma.

## Introduction

Cholangiocarcinoma (CCA) arises from the epithelial cells facing the lumen of the biliary trees and is the second most frequent primary hepatic tumor after hepatocellular carcinoma globally^1,2^. CCA is usually diagnosed at advanced incurable stages due to the absence of prior recognizable clinical manifestations, coupled with the current unavailability of specific tumor biomarkers^3^. Despite the latest progress in the development of molecular targeted therapies, the prognosis for this devastating cancer remains grim^3^. Pemigatinib has recently been approved for 9–14% of CCA patients harboring a fusion or rearrangement of growth factor receptor 2 gene^4^, and ivosidenib has been shown to significantly improve the progression-free survival of patients with isocitrate dehydrogenase-1 mutant advanced CCA in a phase 3 clinical trial^5^. However, CCA is a heterogeneous malignancy and bears a high mutation burden^6^, thus the potential druggable genome alterations in a small proportion of CCAs are not ideal therapeutic targets owing to the anticipated redundancy of signaling pathways^7^. Therefore, there is great urgency in further elucidating the molecular mechanisms and pathways underpinning this disease so that the clinical outcome of CCA patients could be improved.

Neuropilin-1 (NRP-1) is a non-tyrosine kinase transmembrane molecule overexpressed in gastrointestinal cancers^8,9^, and serves as a co-receptor for several cellular signaling pathways involved in cancer progression^10–13^. We have recently demonstrated that human CCA tissues expressed higher levels of NRP-1, which co-activates the vascular endothelial growth factor (VEGF), epidermal growth factor (EGF), and hepatocyte growth factor (HGF)-mediated pathways involved in the progression of CCA^14^. It is known that microRNAs (miRNAs) regulate multiple cellular functions and have emerged as potential targets in anti-cancer campaign^15^. In exploring the miRNA-mediated mechanisms that lead to the overexpression of NRP-1, we have shown that miR-320a negatively regulates NRP-1 by binding to the 3′-UTR of its promoter and is expressed at low levels in CAA tissues and cells^14^. MiR-320a is regarded as a tumor-suppressive miRNA^16^ and inhibits the proliferation and metastasis of CCA cells in vitro and in vivo through downregulating NRP-1^14^. However, its upstream regulatory mechanisms remain unknown.

Long non-coding RNAs (lncRNAs) are a group of non-coding RNAs (ncRNAs) with over 200 nucleotides in length and comprise ~80% of ncRNAs. Emerging studies provide strong evidence that lncRNAs exert pivotal roles in regulating gene expression in many diseases^17^. One of the main regulatory functions of lncRNAs is to act as competing endogenous RNAs (ceRNAs) to sponge miRNAs, leading to the loss of the ability to degrade, silence, or hamper translation of their downstream genes^17^. Many lncRNAs have been shown to regulate key factors involved in cancer cells^18^ and some of them represent potential diagnostic markers and therapeutic targets for CCA^19,20^. Therefore, we carried out the present study to explore potential upstream lncRNAs that can regulate the miR-320a/NRP-1 axis in CCA.

## Results

### Identification of lncRNA TTN-AS1 as a potential target in CCA

The overexpression of NRP-1 in clinical CCA tissues was confirmed by using immunohistochemistry of tissue microarrays (Supplementary Fig. S1). A panel of CCA cell lines expressed different levels of NRP-1, where the order of cell lines with the highest to lowest expression was RBE, HCCC9810, QBC939, CC262, and FRH0201, but all expressed higher levels of NRP-1 than normal human biliary epithelial HIBEC cells (Supplementary Fig. S2A, B). RBE cells expressed the highest levels of NRP-1 protein and mRNA, which were ~7 and ~22 fold higher than HIBEC cells, respectively, and expressed the lowest level of miR-320a, which was 1/10 of that of HIBEC cells (Fig. S2C). A negative correlation was found between expression levels of miR-320a and NRP-1 mRNA (Fig. S2D). LncRNAs that have binding sites with miR-320a were screened by using multiple bioinformatics tools and databases (http://starbase.sysu.edu.cn, DIANA-TarBase, www.lncrnadb.org, LncBase Experimental v.2, lncactdb2.0, https://omictools.com, and http://bioinfo.life.hust.edu.cn), and 10 potential candidates were selected based on the criteria of free energy ≤10 kcal/mol and score >140 (Supplementary Table S1). We then detected their expression levels in RBE and HIBEC cells by quantitative reverse-transcription polymerase chain reaction (qRT-PCR) with specific primers (Supplementary Table S2). Among the 10 candidates, TTN-AS1 (titin-antisense RNA1) was shown to have the largest folds of alteration between RBE and HIBEC cells (Supplementary Fig. S3A, B). Notably, TTN-AS1 is a novel lncRNA derived from the opposite strand of titin (TTN) gene and has partial sequence complementarity with TTN gene^21^. LncRNA TTN-AS1 has been shown to promote the progression of several cancer types including esophageal squamous cell carcinoma (ESCC)^21^, lung adenocarcinoma^22^, and papillary thyroid cancer^23^. The expression of TTN-AS1 was also detected in all the available CCA cell lines and showed a positive correlation with NRP-1, but a negative correlation with miR-320a (Fig. S3C-E).

### Association of TTN-AS1 expression with clinicopathologic parameters of CCA patients

**Fig. 1:**
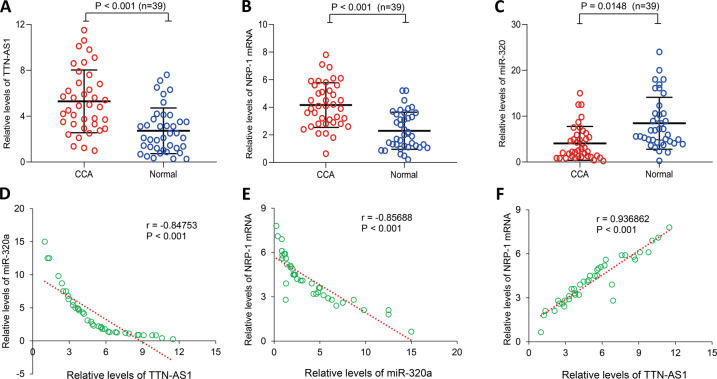
The expression of lncRNA TTN-AS1, miR-320a, and NRP-1 and their correlation in CCA tissues. The expression of TTN-AS1 (**a**), NRP-1 mRNA (**b**), and mature miR-320a (**c**) in 39 pairs of human CCA tissues and corresponding adjacent normal biliary tissues was detected by qRT-PCR. *n*, number of samples examined. Statistical analyses were performed by a Student’s *t* test. **d**–**f** The correlation between miR-320a/TTN-AS1, NRP-1mRNA/miR-320a, and TTN-AS1/NRP-1 mRNA expression was analyzed with a Pearson test.

The qRT-PCR analyses revealed that CCA tumor tissues expressed significantly higher levels of TTN-AS1 (Fig. 1a) and NRP-1 mRNA (Fig. 1b), and significantly lower levels of miR-320a (Fig. 1c), compared with adjacent normal bile duct tissues. In CCA tissues, an inverse correlation between expression levels of TTN-AS1 and miR-320a (Fig. 1d) and between miR-320a and NRP-1 mRNA (Fig. 1e) and a positive correlation between TTN-AS1 and NRP-1 mRNA (Fig. 1f) were found by using Pearson correlation analyses. Based on the expression levels of TTN-AS1, we divided 39 CCA cases into the high (>mean) and low (≤mean) groups, and analyzed the association between TTN-AS1 expression and clinicopathologic parameters. The results showed that the expression of TTN-AS1 was significantly correlated with tumor differentiation and lymph node metastasis, and marginally correlated with portal vein invasion, while not with gender, age, tumor location, or TNM staging (Table 1). Namely, CCA patients with poor tumor cell differentiation, positive lymph metastasis, and portal vein invasion had higher expression of TTN-AS1 (Table 1). By using the same analyses based on expression levels of NRP-1 mRNA and miR-320a, we found that both were correlated with tumor differentiation, lymph node metastasis, and portal vein invasion. Further, NRP-1 mRNA expression levels also correlated with TNM staging (Table 1).

**Table 1:** Correlations of the expression of TTN-AS1, NRP-1 mRNA, or miR-320a with clinicopathological parameters of CCA patients.

Parameters	Total (*n* = 39)	TTN-AS1		*P* value	NRP-1 mRNA		*P* value	miR-320a		*P* value
		Low (*n* = 18)	High (*n* = 21)		Low (*n* = 21)	High (*n* = 18)		Low (*n* = 22)	High (*n* = 17)	
Gender				0.9595			0.4771			0.3072
Male	24	11	13		14	10		12	12	
Female	15	7	8		7	8		10	5	
Age (years)				0.2042			0.4956			0.6479
<60	26	13	11		13	13		14	12	
≥60	13	5	10		8	5		8	5	
Tumor differentiation				0.0042^a^			0.01823^a^			0.009076^a^
Well/moderate	23	15	8		16	7		9	14	
Poor	16	3	13		5	11		13	3	
Tumor location				0.7079			0.7481			0.9681
Intrahepatic	12	5	7		6	6		8	4	
Perihilar	27	13	14		15	12		14	13	
Tumor size				0.137			0.6555			0.06524
< 50 mm	21	12	9		12	9		9	12	
≥ 50 mm	18	6	12		9	9		13	5	
TNM stage^b^				0.1517			0.01543^a^			0.267
I/II	19	11	8		14	5		9	10	
III/IV	20	7	13		7	13		13	7	
Lymph metastasis				0.0182^a^			0.02709^a^			0.0194^a^
Negative	16	11	5		12	4		6	11	
Positive	23	7	16		9	14		16	6	
Portal vein invasion				0.0828			0.03307^a^			0.04106^a^
No	18	11	7		13	5		7	11	
Yes	21	7	14		8	13		15	6	

^a^indicates a significant difference.

^b^According to the 8th UICC (Union for International Cancer Control)-TNM staging system. *P* value was estimated by a *χ*^2^ test.

*CCA* cholangiocarcinoma, *TTN-AS1* lncRNA titin-antisense RNA1, *NRP-1* neuropilin-1.

### TTN-AS1 functions as a ceRNA to sponge miR-320a

For examining the regulatory effects between TTN-AS1 and miR-320a, we first showed that transfection of miR-320a mimics had little effect on TTN-AS1 expression, but depletion of TTN-AS1 significantly increased the expression of miR-320a in RBE and HCCC9810 cells (Supplementary Fig. S4A, B), implying that TTN-AS1 might negatively regulate miR-320 in CCA cells. Based on the putative binding sites between TTN-AS1 and miR-320a, luciferase reporter and RNA pulldown assays were employed to examine their direct binding (Supplementary Fig. S5). The luciferase intensity was decreased by co-transfected miR-320a mimics and wild-type TTN-AS1 reporter vector but not the mutant reporter vector lacking the miR-320a binding site. Consistently, miR-320a was precipitated by wild-type TTN-AS1 but not TTN-AS1 mutant; and TTN-AS1 was pulled down by biotin-labeled wild-type miR-320a but not miR-320a mutant (Fig. S5).

### TTN-AS1 regulates miR-320a in an argonaute2-dependent manner

**Fig. 2:**
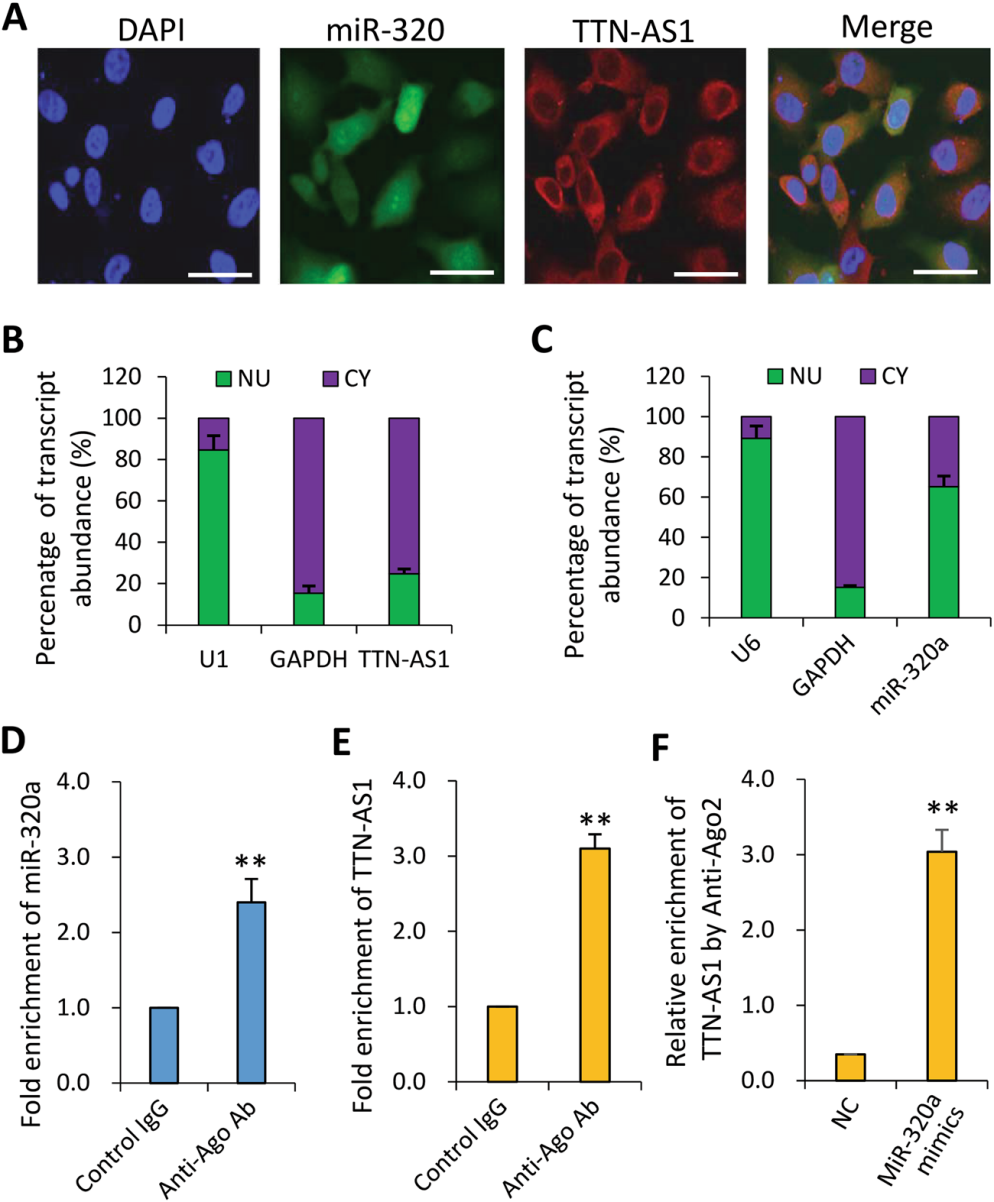
TTN-AS1 regulates the expression of miR-320a in the cytoplasm of CCA cells in an Ago2-dependent manner. **a** RBE cells were subjected to in situ hybridizations of miR-320a (5′-DIG tagged probe, identified with Cy3-conjugated Ab in red) and TTN-AS1 (5′-DIG tagged probe, identified with FITC-conjugated Ab in green), and stained with DAPI (blue). Three images from the same cells were merged. Scale bar, 25 μm. **b**, **c** Total RNA was extracted from nuclear (NU) and cytoplasmic (CY) fractions of RBE cells, and the expression of TTN-AS1 (**b**) and miR-320a (**c**) was measured by qRT-PCR and normalized. U1 and U6 were used as internal nuclear controls for TTN-AS1 and miR-320a, respectively, and GAPDH as an internal cytoplasmic control. **d**, **e** RBE cells were subjected to RNA immunoprecipitation (RIP) assays. The fold enrichment of miR-320a (**d**) and TTN-AS1 (**e**) by an anti-Ago2 Ab was normalized to a nonspecific IgG acting as a negative control. **f** RBE cells transfected with negative control (NC) or miR-320a mimics were subjected to RIP to measure relative enrichment of TTN-AS1 by the anti-Ago2 Ab. **P < 0.001 indicates a significant difference from respective controls.

The above results indicate that miR-320a binds to lncRNA-TTN-AS1 without causing TTN-AS-1 degradation. TTN-AS1 and miR-320a were both located in the cytoplasm of CCA cells as detected by In situ hybridization (Fig. 2a–c), suggesting that TTN-AS1 may bind to miR-320a through the argonaute2 (Ago2)-dependent RNA interference pathway^24^. As expected, RNA immunoprecipitation (RIP) assay showed levels of miR-320a and TTN-AS1 precipitated by an anti-Ago2 Ab were markedly increased, resulting in a ~2 and ~3-fold enrichment compared with control IgG, respectively (Fig. 2d, e). Meanwhile, endogenous TTN-AS1 pulldown by the anti-Ago2 Ab was specifically enriched upon ectopic overexpression of miR-320a (Fig. 2f). These data suggest that TTN-AS1 binds to miR-320a in the cytoplasm in an Ago2-dependent manner.

In addition, the expression of miR-320a was downregulated by TTN-AS1 overexpression and upregulated by TTN-AS1 knockdown, and these effects could be abolished by miR-320a mimics and antagomiR-320a, respectively (Supplementary Fig. S6A, B). However, no significant difference in TTN-AS1 expression was detected by transfection of miR-320a mimics or antagomiR-320a (Fig. S6C, D). These results indicate that the regulatory effects between TTN-AS1 and miR-320a are in a one-way manner.

### TTN-AS1 promotes the proliferation of CCA cells via miR-320a/NRP-1

We have previously reported that NRP-1 depletion and ectopic expression of miR-320a inhibited the proliferation of CCA cells^14^. In accord, we confirmed that depletion of NRP-1 significantly reduced cell viability, while miR-320a mimics showed a similar effect by downregulating NRP-1 expression (Supplementary Fig. S7). We could further show that knockdown of TTN-AS1 significantly reduced cell viability while antagomiR-320a partially restored cell viability (Supplementary Fig. S8A). Mechanistically, TTN-AS1 knockdown led to a significant downregulation of NRP-1, cyclin-dependent kinase 2 (CDK2) and cyclin E, a significant upregulation of p27, but had little effect on the expression of cyclin D1 and p21. The above molecules are key factors involved in cell proliferation and cycle progression^25^. AntagomiR-320a counteracted the effect of TTN-AS1 knockdown (Fig. S8B). Cell cycle distribution assays showed that knockdown of TTN-AS1 led to more cells arrested at the G0/G1 phase, while antagomiR-320a partially abolished this effect of TTN-AS1 knockdown (Supplementary Fig. S9).

On the other hand, exogenous overexpression of TTN-AS1 increased the viability of FRH0201 cells, while miR-320a mimics partially abolished this effect (Fig. S8C). TTN-AS1 overexpression resulted in the upregulation of NRP-1, cyclin E, and CDK2, and downregulation of p27; while miR-320a mimics could neutralize the effect of TTN-AS1 overexpression (Fig. S8D).

### TTN-AS1 promotes the migration of CCA cells via the miR-320a/NRP-1 axis

**Fig. 3:**
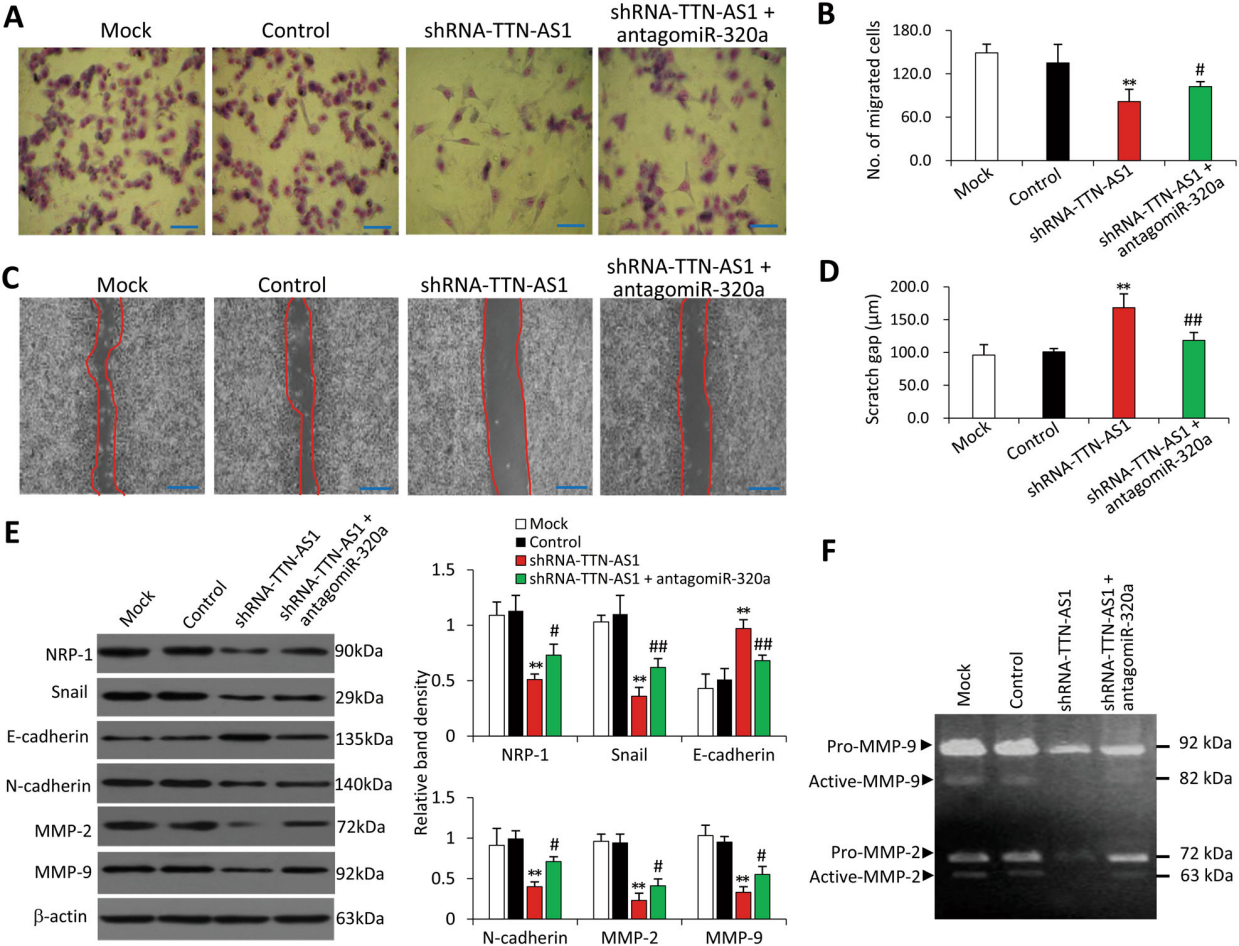
Knockdown of TTN-AS1 inhibits cell migration via regulating miR-320a. RBE cells were transfected with scrambled shRNA (control), shRNA-TTN-AS1, or shRNA-TTN-AS1 + antagomiR-320a for 48 h. Cells were subjected to transwell migration (**a**, **b**) and scratch (**c**, **d**) assays. **a** Migrated cells were visualized using Giemsa staining. Scale bar, 50 μm (**a**) and 200 µm (**c**). **b** Numbers of migrating cells were counted. **c** Scratch areas were recorded. **d** Scratch distances were quantified at indicated time points. **e**, **f** Cells were immunoblotted for detecting key EMT proteins and the density of each band was normalized to β-actin. **g** Cells were subjected to gelatin zymography assays for analyzing the gelatinolytic activity of MMP-9 and MMP-2. **P < 0.001 vs. controls, and ^#^*P* < 0.05 and ^##^*P* < 0.001 vs. shRNA-TTN-AS1.

Knockdown of TTN-AS1 significantly reduced the ability of RBE cells to migrate, while antagomiR-320a partially abolished this effect (Fig. 3a–d). CCA cells acquire the migratory and invasive properties through a critical process known as epithelial-mesenchymal transition (EMT)^26^. Therefore, we examined the effects of TTN-AS1 knockdown on the expression of decisive factors involved in the process of EMT^27^. TTN-AS1 knockdown significantly downregulated the expression of NRP-1, Snail, N-cadherin, matrix metalloproteinase (MMP)-2, and MMP-9, and upregulated the expression of E-cadherin (Fig. 3e). The results were supported by gelatin zymography assays, which showed that TTN-AS1 knockdown significantly reduced activities of MMP-2 and MMP-9, while antagomiR-320a partially counteracted this effect (Fig. 3f). On the other hand, overexpression of TTN-AS1 increased the migratory ability of FRH0201 cells while miR-320a mimics partially abolished this effect (Supplementary Fig. S10).

### TTN-AS1 contributes to the growth of CCA tumors in animal models

**Fig. 4:**
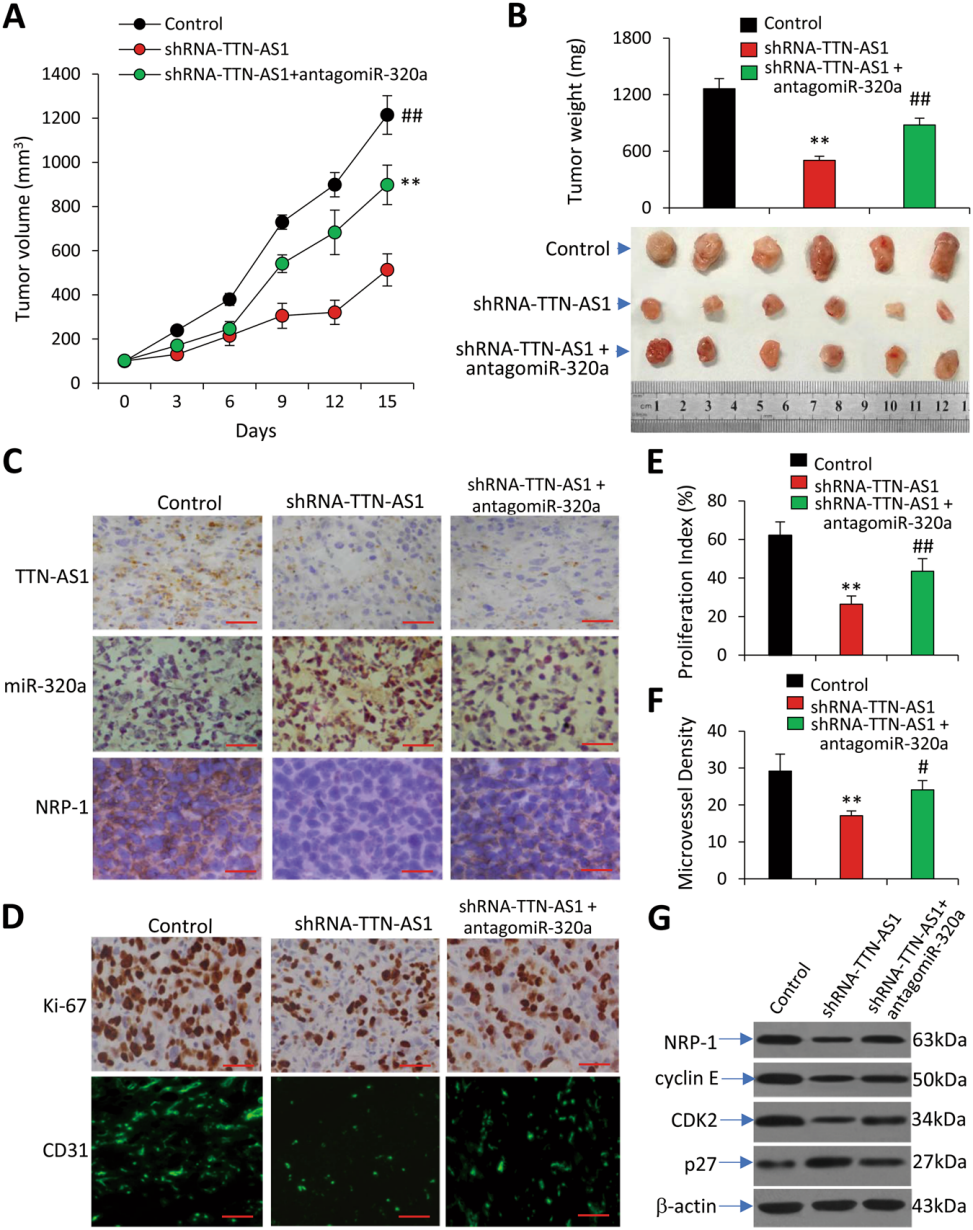
Knockdown of TTN-AS1 inhibits the growth and angiogenesis of CCA tumors in vivo. Subcutaneous CCA tumors were established in mice by inoculation of RBE cells and received respective treatments as described in Supplementary Information. **a** The growth curve of RBE tumors was recorded. **b** RBE tumors were resected, weighed, and photographed at the end of experiments. **c** Two mice were killed from each group to harvest tumors 2 days after treatments and the expression of TTN-AS1 and miR-320a was examined by in situ hybridization (magnification ×200; Scale bar, 100 μm), and NRP-1 expression by immunohistochemistry (Magnification ×400; Scale bar, 50 μm). Tumors harvested at the end of experiments. **d** Illustrated are representative tumor sections immunostained by Abs against Ki-67 and CD31, respectively (Magnification ×400; Scale bar, 75 μm). In situ cell proliferation index (**e**) and tumoral microvessel density (**f**) were quantified. **g** Tumor tissue homogenates were immunoblotted for detecting the expression of key proliferation proteins. **P < 0.001 vs. controls; ^#^*P* < 0.05 and ^##^*P* < 0.001 vs. shRNA-TTN-AS1.

The functional role of TTN-AS1 was also confirmed in CCA tumors in vivo. Subcutaneous RBE tumors were established in mice, which were randomly assigned to different treatments when tumors reached ~100 mm^3^. Tumors treated with shRNA-TTN-AS1 were significantly smaller (513.2 ± 72.7 mm^3^) than control tumors (1214.9 ± 90.3 mm^3^), however, co-treatment of antagomiR-320a could partially restore the growth of tumors (903.6 ± 88.4 mm^3^), as measured 15 days after treatment commencement (Fig. 4a). The results of tumor volume correlated with the weight of tumors (Fig. 4b). Treatment of shRNA-TTN-AS1 led to TTN-AS1 downregulation and miR-320a upregulation in tumors harvested 2 days after treatments by in situ hybridization, and downregulation of NRP-1 by immunohistochemistry (Fig. 4c). Co-treatment of antagomiR-320a partially abolished the effects of shRNA-TTN-AS1 on miR-320a upregulation and NRP-1 downregulation but had little effect on TTN-AS1 expression (Fig. 4c). Treatment of shRNA-TTN-AS1 significantly inhibited cell proliferation in situ (Fig. 4d, e) and reduced tumor vasculature, while antagomiR-320a neutralized the effects of shRNA-TTN-AS1 (Fig. 4d, f). In agreement with the in vitro results (Fig. S8A, B), immunoblotting analysis of tumor homogenates showed that shRNA-TTN-AS1 treatment led to downregulation of NRP-1, cyclin E, and CDK2, and upregulation of p27; while antagomiR-320a counteracted the effects of shRNA-TTN-AS1 (Fig. 4g).

On the other hand, by adopting another subcutaneous CCA tumor mouse model with FRH0201 cells, which were shown to express a lower level of TTN-AS1 (Fig. S2A), we demonstrated that exogenous overexpression of TTN-AS1 promoted tumor growth by promoting in situ cell proliferation and tumor angiogenesis, while miR-320a mimics partially abolished these effects (Supplementary Fig. S11).

### TTN-AS1 regulates the c-Met and TGF-β pathways via NRP-1

**Fig. 5:**
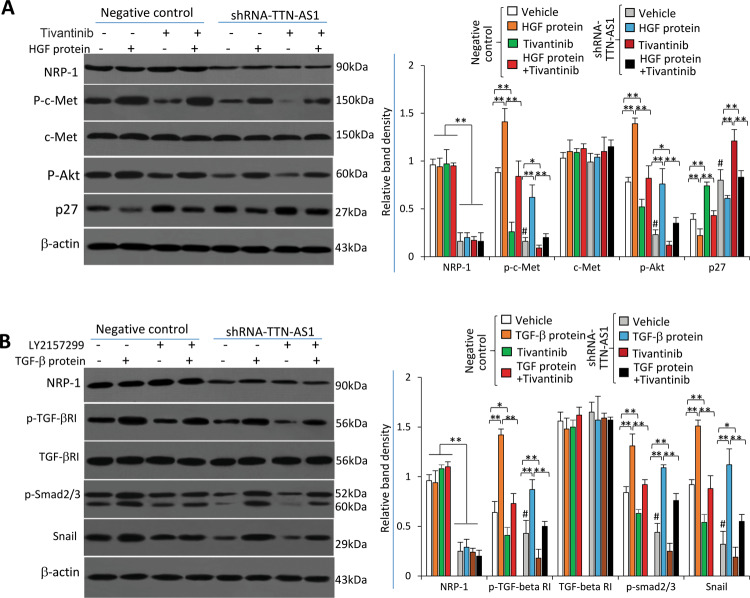
TTN-AS1 regulates the c-Met and TGF-β pathways via NRP-1. RBE and cells were transfected with negative control or shRNA-TTN-AS1, and then incubated for 24 h in the presence or absence of recombinant HGF protein (100 ng/ml) and tivantinib (10 μg/ml) (**a**), or TGF-β protein (5 ng/ml) and LY2157299 (10 μg/ml) (**b**). Cell lysates were immunoblotted to determine the expression of key proteins involved in the above pathways as indicated. The density of each band was normalized to β-actin. *P < 0.05 and **P < 0.001 indicate a significant difference. ^#^*P* < 0.001 indicates a significant difference from negative control cells treated with vehicle.

We have previously demonstrated that NRP-1 co-activates the HGF/c-Met pathway in CCA cells^14^. Control and shRNA-TTN-AS1-transfected RBE cells were incubated with recombinant human HGF protein in the presence or absence of tivantinib, a c-Met inhibitor and an anti-cancer drug used in CCA clinical trial^28^. TTN-AS1 knockdown led to downregulation of NRP-1 expression, resulting in downregulation of phosphorylated c-Met (p-c-Met), and sequential downregulation of phosphorylated Akt (p-Akt) and upregulation of p27 (Fig. 5a). Incubation of HGF protein did not affect NRP-1 expression but could activate the c-Met pathway, evidenced by upregulation of p-c-Met and p-Akt, and downregulation of p27; and incubation of tivantinib showed the opposite effects to HGF ligand (Fig. 5a).

NRP-1 co-interacts transforming growth factor (TGF)-β pathway^29^, which is crucial for EMT of cancer cells^30^. Therefore, we examined the effect of TTN-AS1 knockdown on this pathway in CCA cells. Control and shRNA-TTN-AS1-transfected RBE cells were incubated with recombinant human TGF-β protein or/and LY2157299, a specific TGF-β receptor (TGF-βR) inhibitor^31^. Incubation of TGF-β protein or LY2157299 did not affect the expression of NRP-1 or TGF-βRI (Fig. 5b). However, TGF-β induced the upregulation, while LY2157299 reduced the expression, of p-TGF-βRI. TTN-AS1 knockdown had little effect on TGF-βRI expression, but significantly inhibited its phosphorylation (Fig. 5b). The activation of TGF-β pathway by TGF-β protein increased the sequential expression of p-Smad2/3 and Snail, while LY2157299 and TTN-AS1 knockdown demonstrated opposite effects and abolished the activating effects of TGF-β protein (Fig. 5b).

## Discussion

**Fig. 6:**
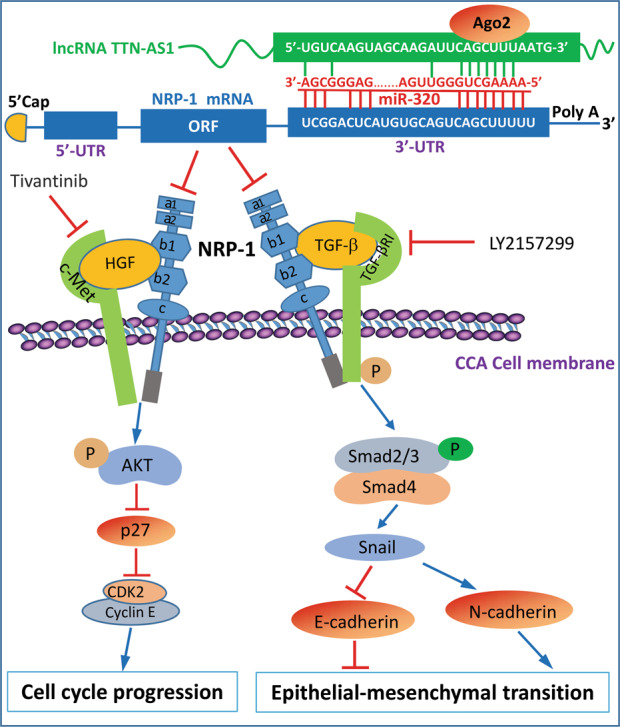
Schematic diagram of the TTN-AS1/miR-320a/NRP-1 axis contributing to the progression of CCA. LncRNA TTN-AS1 serves as a ceRNA to sponge miR-320a through complementary binding sites in an Ago2-dependent manner in CCA cells. On the other hand, miR-320a downregulates the expression of NRP-1 by binding to its 3′-UTR. An NRP-1 protein molecule is composed of five extracellular domains (a1, a2, b1, b2, and c), one transmembrane domain and a short cytosolic tail, and acts as a co-receptor for ligands (HGF and TGF-β) to stimulate the activation of respective c-Met and TGF-β signaling pathways. “→” indicates promotion, positive regulation, or activation; “⊥” indicates inhibition, negative regulation, or blockade. “p” indicates phosphorylation of proteins. Ago2 argonaute2, CCA cholangiocarcinoma, CDK2 cyclin-dependent kinase 2, HGF hepatocyte growth factor, NRP-1 neuropilin-1, ORF open reading frame, TGF-β transforming growth factor-β, TGF-βR TGF-β receptor, UTR untranslated region.

LncRNA TTN-AS1 was initially reported to participate in the progression and metastasis of ESCC^21^. Later, its functional role was confirmed in other cancer types^22,23,32,33^. Importantly, TTN-AS1 exerts regulatory effects via acting as a ceRNA to sponge different miRNAs in different cancers. For instance, TTN-AS1 regulated the miR-133b/actin-binding protein fascin homolog 1 axis in ESCC cells^21^, while promoted the migration and EMT of lung adenocarcinoma cells by sponging miR-142-5p to regulate CDK5^22^. As schematically summarized in Fig. 6, we have, in the present study, found that TTN-AS1 serves as a ceRNA to sponge miR-320a through complementary binding sites in an Ago2-dependent manner in CCA cells.

LncRNAs exhibit different functions depending on their subcellular localization. This study showed that TTN-AS1 was mainly localized in the cytoplasm of CCA cells, while miR-320a was located in both nuclear and cytoplasm subcellular compartments. It is well established that the maturation of miRNAs occurs in the cytoplasm, where they execute post-transcriptional gene silencing via an RNA-induced silencing complex pathway^34^. Intriguingly, the ectopic expression of miR-320a reduced the luciferase activities of the wild-type TTN-AS1 reporter, but the expression of TTN-AS1 remained unchanged upon overexpression of miR-320a. Moreover, endogenous TTN-AS1 and miR-320a could be pulled down by an anti-Ago2 Ab. These data suggest that miR-320a recognizes and binds with TTN-AS1 without triggering the degradation of TTN-AS1, which plays a post-transcriptional regulatory role in downregulating miR-320a via an Ago2-dependent way and in a one-way manner in CCA cells. In accord, it has been reported that TTN-AS1 was located mainly in the cytoplasm and acted as a ceRNA sponging miRNAs in ESCC and papillary thyroid cancer cells^21,23^.

MiR-320a is one of the two most highly downregulated miRNAs in clinical CCA tissues and is closely associated with the progression and severity of CCA^35^. MiR-320a also represents a critical suppressor component of the progression of other cancers^16,36,37^. We have previously reported that miR-320a negatively regulated the expression of NRP-1 by binding to the 3′-UTR of NRP-1 promoter, and inhibited cell proliferation and migration of CCA cells^14^.

NRP-1 functions as versatile co-receptors that bind to a number of growth factors and couple with cognate receptor tyrosine kinase signaling pathways involved in cancer progression^11,14,38^. In the present study, we have further demonstrated that NRP-1 acts as a co-receptor for the activation of HGF/c-Met pathway, which induces the phosphorylation of Akt^39^, a downstream of c-Met signaling^40^. Akt activation leads to the sequential downregulation of p27^41^, which inactivates the CDK2/cyclin E complex, resulting in cell cycle arrest^41^. However, tivantinib, a specific c-Met inhibitor can block NRP-1-induced activation of the HGF/c-Met pathway (Fig. 6). On the other hand, NRP-1 co-interacts with TGF-β^29^, leading to the activation of the TGF-β/TGF-βRI pathway, which in turn increases the expression of phosphorylated Smad2 and Smad3. The latter two combine with Smad4 to form a trimeric SMAD complex that upregulates the expression of Snail, which conveys TGF-β-induced repression of E-cadherin and stimulation of N-cadherin^42^, thus promoting EMT of CCA cells. However, LY2157299, a specific TGF-βR inhibitor^31^, can block NRP-1-induced activation of the TGF-β/TGF-βRI pathway (Fig. 6). As demonstrated previously^14^, but not investigated in this study, the above signaling pathways may also cross-talk with each other and contribute to the proliferation and metastasis of cancer cells^43^.

In summary, to the best of our knowledge, this is the first study that reports the functional role of TTN-AS1 as a sponging ceRNA for miR-320a, its high expression in CCA tissues and a significant association with clinicopathologic parameters of CCA. TTN-AS1 displays its regulatory activity by binding to miR-320a through the Ago2-dependent RNA interference pathway and in a one-way manner in the cytoplasm of CCA cells. Through downregulating miR-320a, TTA-AS1 promotes cell cycle progression, EMT, and angiogenesis via NRP-1, which co-interacts HGF/c-Met and TGF-β/TGF-βRI pathways in CCA cells. Taken together, the present study has unveiled a novel axis consisting of TTN-AS1/miR-320a/NRP-1, which may also represent a therapeutic target and biomarkers in the management of CCA.

## Materials and methods

### Clinical CCA tissues

A total of 39 pairs of CCA and matched adjacent normal bile duct tissues were collected at the Department of Hepatobiliary Surgery, Shandong Provincial Hospital. Among them, 23 pairs have been described previously^14^ while 16 new pairs of tissues were collected between April, 2018 and September, 2019. Of the 39 cases, 27 were perihilar CCA and 12 were intrahepatic CCA. The criteria of the included specimens were consistent with our previous study^14^. The current study has been approved by the Ethics Committee of Shandong Provincial Hospital (Jinan, China) and informed consent was obtained from all subjects.

### Cells, antibodies, and reagents

Human CCA cell lines (HCCC9810, RBE, QBC939, CC262, and FRH0201) and normal human biliary epithelial HIBEC cells were obtained from the Cell Bank of the Chinese Academy of Sciences (Shanghai, China)^14,44^. Cells were routinely cultured at 37 °C in RPMI-1640 medium supplemented with 10% (v/v) fetal bovine serum in a humidified atmosphere of 5% CO_2_. Cell lines were confirmed to be negative for mycoplasma infection by using a PCR-based Universal Mycoplasma Detection kit (American Type Culture Collection, Manassas, VA, USA). Relevant information regarding antibodies (Abs), reagents, and kits are described in detail in Supplementary Information.

### Animal experiments

The experimental protocol has been described previously^14,44^ and approved (permit SYXK20020009) by the Institute Animal Ethics Committee. Immunodeficient nude BALB/c mice (H-2b) were housed in the Animal Research Center, the First Affiliated Hospital of Harbin Medical University, China. Two sets of experiments were designed to examine the effects of TTN-AS1 knockdown and overexpression on tumor growth. Detailed information for animal experiments is included in Supplementary Information. Briefly, cells were injected subcutaneously into mice and palpable tumors were monitored. Around 2–3 weeks later, mice bearing tumors with a volume of ∼100 mm^3^ were randomly assigned to different groups (*n* = 8). The TTN-AS1 knockdown study had three groups of animals, which received intratumoral injections of control, shRNA-TTN-AS1 or shRNA-TTN-AS1 + antagomiR-320a, respectively; while the TTN-AS1 overexpression study comprised three groups of animals, which received injections of either control, TTN-AS1 or TTN-AS1 + miR-320a mimics, respectively. Two mice from each group were killed 2 days after injection for detecting gene expression. The remaining mice were further monitored and euthanized 15 days after treatments commenced.

Immunohistochemistry, Tissue microarrays, Establishment of stable transfectants depleted of NRP-1, Assays of cell viability, cell cycle, Transwell migration, Cell scratch, qRT-PCR, western blot and Gelatin zymography, Cell fraction isolation, In situ hybridization, RNA pulldown and RIP assays, Transfection of miR-320a mimics, antagomiR-320a and TTN-AS1 expression vectors, Plasmid constructs and luciferase assay, In situ Ki-67 proliferation index, and Assessment of tumor vascularity.

The detailed description for these methods is included in Supplementary Information and has also been described previously^11,14^.

### Statistical analysis

GraphPad Prism 8.02 (GraphPad Software, San Diego, CA, USA) was employed for performing statistical analyses. Data are expressed as mean values ± standard deviation. Multiple comparisons were made with a one-way analysis of variance (ANOVA) followed by a Tukey post-hoc test. Comparisons between two groups were made by a *t*-test. Correlations of TTN-AS1, NRP-1 mRNA, or miR-320a with clinicopathological parameters were estimated by a *χ*^2^ test. The relationship between two variables was analyzed by using Pearson’s correlation coefficient. *P* < 0.05 was considered statistically significant.

## Supplementary information

Supplementary Information

Supplementary Figure Legends

Supplementary Fig. S1

Supplementary Fig. S2

Supplementary Fig. S3

Supplementary Fig. S4

Supplementary Fig. S5

Supplementary Fig. S6

Supplementary Fig. S7

Supplementary Fig. S8

Supplementary Fig. S9

Supplementary Fig. S10

Supplementary Fig. S11
