# Supplementary material for: LncRNA TTN-AS1 promotes the progression of cholangiocarcinoma via the miR-320a/neuropilin-1 axis
Source: Cell Death Dis. 2020 Aug 15;11(8):637. doi: 10.1038/s41419-020-02896-x (PMC7429853; doi:10.1038/s41419-020-02896-x)
Supplement: Supplementary file 1 — Supplementary Information [file 41419_2020_2896_MOESM1_ESM.docx]

**Supplementary Information for**

**LncRNA TTN-AS1 promotes the progression of cholangiocarcinoma via the miR-320a/neuropilin-1 axis**

Huaqiang Zhu^1^, Bo Zhai^2,3^, Changjun He^4^, Ziyi Li^2^, Hengjun Gao^1^, Zheyu Niu^1^, Xian Jiang^2^, Jun Lu^1,*^, Xueying Sun^2,*^

^1^Department of Hepatobiliary Surgery, Shandong Provincial Hospital Affiliated to Shandong First Medical University, Jinan 250021, China

^2^ The Hepatosplenic Surgery Center, the First Affiliated Hospital of Harbin Medical University, Harbin 150001, China

^3^ Department of General Surgery, the Fourth Affiliated Hospital of Harbin Medical University, Harbin 150001, China

^4^ Department of Surgery, the Third Affiliated Hospital of Harbin Medical University, Harbin, China.

**Contents**

**Supplementary Materials and Methods** ................................................................ 4

List of antibodies, main reagents and kits………………………….......................4

Immunohistochemistry for clinical specimens…....................................................5

Tissue microarrays...….............................................................................................6

Establishment of stable transfectants depleted of NRP-1........................................7

Cell viability analysis...............................................................................................7

Cell fraction isolation……………………………...................................................8

In situ hybridization for detecting miR-320a and TTN-AS1………………………8

RNA pulldown and RNA immunoprecipitation (RIP) assays...................................9

Transfection of oligonucleotides targeting miR-320a and TTN-AS1

expression vectors....................................................................................................10

Assessment of cell cycle……………………………….………………………......11

Cell scratch assay…….............................................................................................11

Transwell migration assay………...........................................................................12

Quantitative reverse-transcription polymerase chain reaction (qRT-PCR) .............12

Luciferase assays and plasmid constructs...…………………………………….…13

Western blot analysis...............................................................................................14

Gelatin zymography assay.......................................................................................14

Animal experiments.................................................................................................15

Immunohistochemistry of assessing gene expression in animal tumor tissues.......16

*In situ* Ki-67 proliferation index..............................................................................17

Assessment of tumor vascularity.............................................................................17

**Supplementary Tables…**...........................................................................................17

Table. S1..................................................................................................................18

Table S2...................................................................................................................21

**References**..................................................................................................................21

**List of antibodies, main reagents and kits used in the study**

| Name | Catalogue No. | Supplier |
| --- | --- | --- |
| Anti-Snail Ab | #3879 | CST |
| Anti-E-Cadherin Ab | #3195 | CST |
| Anti-N-Cadherin Ab | #13116 | CST |
| Anti-Smad2/3 Ab | #8685 | CST |
| Anti-phosphor-Smad2 (Ser465/467)/ Smad3 (Ser423/425) Ab | #9510 | CST |
| Anti-Smad4 Ab | #38454 | CST |
| Anti-p27 Ab | #3688 | CST |
| Anti-cyclin D1 Ab | #2922 | CST |
| Anti-Akt Ab | #4691 | CST |
| Anti-phosphor-Akt (Ser473) Ab | #4060 | CST |
| Anti-β-actin Ab | sc-130065 | Santa Cruz |
| FITC-conjugated rat anti-mouse Ab | sc-516140 | Santa Cruz |
| Anti-β-actin Ab | sc-130065 | Santa Cruz |
| Anti-NRP-1 Ab | sc-5307 | Santa Cruz |
| Anti-cyclin E Ab | sc-247 | Santa Cruz |
| Anti-CDK2 Ab | sc-6248 | Santa Cruz |
| Anti-p21 Ab | sc-53870 | Santa Cruz |
| Anti-MMP-2 | sc-13594 | Santa Cruz |
| Anti-MMP-9 | sc-21733 | Santa Cruz |
| Anti-Ki67 Ab | ab15580 | Abcam |
| Anti-CD31 Ab | ab28364 | Abcam |
| Anti-c-Met Ab | ab51067 | Abcam |
| Anti- phosphor-c-Met (Thr1349) Ab | ab68141 | Abcam |
| Anti-TGF-βR I Ab | ab31013 | Abcam |
| Anti-phosphor- TGF-βRI  (Ser165) Ab | ab112095 | Abcam |
| Recombinant human HGF protein | PHG0321 | ThermoFisher |
| Recombinant human TGF-βR 1 protein | ab50036 | Abcam |
| Anti-Argonaute2 Ab | ab32381 | Abcam |
| Mouse anti-Digoxigenin Ab | ab420 | Abcam |
| Cy3-conjugated goat anti-mouse Ab | ab97035 | Abcam |
| HRP-conjugated goat anti-mouse | ab6789 | Abcam |
| Anti-Ki67 Ab | ab15580 | Abcam |
| HRP goat anti-mouse Ab | TA130004 | OriGene |
| HRP goat anti-rabbit Ab | TA140003 | OriGene |
| HRP rabbit anti-goat Ab | TA130032 | OriGene |
| HRP rabbit anti-rat Ab | TA130038 | OriGene |
| VECTASTAIN® ELITE ABC kit | SK-4100 | Vector |
| TUNEL kit | # 11684795910 | Sigma-Aldrich |
| Lipofectamine2000 | #11668019 | Invitrogen |
| CCK-8 | CK04-05 | Dojindo |
| DAPI | d9564 | Sigma-Aldrich |
| TRIzol™ Reagent | 15596026 | ThermoFisher |
| LY2157299 | S2230 | Selleckchem |
| tivantinib | S2753 | Selleckchem |
| Lipofectamine2000 | #11668019 | Invitrogen |

Notes: Ab, antibody; BosterBio, Boster Biological Technology (Pleasanton, CA, USA); CST, Cell Signaling Technology (Boston, MA, USA); Santa Cruz; Santa Cruz Biotechnology (Santa Cruz, CA, USA); Cytoskeleton (Denver, CO, USA); R&D Systems (Minneapolis, MN, USA); Sigma-Aldrich (St. Louis, MO, USA); Invitrogen (Carlsbad, CA, USA), FITC, fluorescein isothiocyanate; HRP, horseradish peroxidase; OriGene (OriGene Technologies, Inc., Beijing, China); S6K (ribosomal protein S6 kinase); TUNEL, Terminal deoxynucleotidyl transferase-mediated dUTP nick end labeling agent; Dojindo, Dojindo Molecular Technologies, Gaithersburg, MD, USA; Vector, Vector Laboratories (CA, USA); DAPI, 4',6-diamidino-2-phenylindole, GSK-3β, glycogen synthase kinase 3β; GAPDH, glyceraldehyde 3-phosphate dehydrogenase; mTOR, mammalian target of rapamycin; PTEN, phosphatase and tensin homolog; 4EBP1, eukaryotic translation initiation factor 4E-binding protein 1; LC3, microtubule-associated protein 1 light chain 3. Note: Ab, primary antibody; CST, Cell Signaling Technology (Boston, MA, USA); Santa Cruz; Santa Cruz Biotechnology (Santa Cruz, CA, USA); Cytoskeleton (Denver, CO, USA); R&D Systems (Minneapolis, MN, USA); Sigma-Aldrich (St. Louis, MO, USA); Invitrogen (Carlsbad, CA, USA), MMP-2, matrix metalloproteinase-2; MMP-9, matrix metalloproteinase-9; FITC, fluorescein isothiocyanate; HRP, horseradish peroxidase; OriGene (OriGene Technologies, Inc., Beijing, China); TUNEL, Terminal deoxynucleotidyl transferase-mediated dUTP nick end labeling agent; Dojindo, Dojindo Molecular Technologies, Gaithersburg, MD, USA; Vector, Vector Laboratories (CA, USA); DAPI, 4',6-diamidino-2-phenylindole.

**Immunohistochemistry of clinical specimens**

Formalin-fixed specimens were transferred to 70% ethanol, and subsequently paraffin-embedded, sectioned and mounted on 3-aminopropyltriethoxysilane-coated slides (Sigma, Shanghai, China). Antigen retrieval was performed by heating sections in a microwave in 0.01 M citrate buffer. Sections were blocked for 2 h and incubated with a rabbit anti-human NRP-1 Ab (diluted 1:250) at 4^o^C overnight. A standard horseradish peroxidase staining procedure was followed using a biotinylated secondary Ab （diluted 1:250, and immunoreactivity developed with Sigma FAST DAB (3,3’-diaminobenzidine tetrahydrochloride) and CoCl_2_ enhancer tablets. Sections were counterstained with hematoxylin. Normal rabbit sera were diluted 1:10 in PBS, and used for blocking and dilution of Abs. Negative controls were achieved by using irrelevant goat IgG at a dilution of 1:50. NRP-1 staining was assessed in 20 randomly selected fields per specimen using a semi-quantitative grading system, which reflected the proportion and intensity of staining present within the specimen. The staining intensity (Value A) was graded in a four-tier grading system: no staining (0), faint yellow (1), yellow (2) and brown (3). The extent of positive staining (Value B) was determined using a four-tier grading system based on the percentage of positive cells: ≤ 10% (1), 11-40% (2), 41-70% (3), and ≥ 70% (5). The immunohistological score for each specimen was calculated by A × B, and then each specimen was graded lower level (≤ 5) and higher level (>5).

**Tissue microarrays**

Paraffin-embedded CCA and normal tissue samples were first stained with hematoxylin and eosin (HE). One representative region (2 mm × 2 mm) was selected from each sample and perforated to prepare tissue microarrays (Shanghai Outdo Biotech Co., Ltd., China) as described previously^1^. Tissue microarrays were deparaffinized and dehydrated with graded alcohol, and then subjected to immunohistochemistry.

**Establishment of stable transfectants depleted of NRP-1**

The NRP-1 shRNA pSuppressorNeo vector targeting NRP-1 gene sequence (GAGAGGUCCUGAAUGUUCC) (corresponding to nucleotides 949-967 of human NRP-1 [GenBank NM_003873.5]) and a scrambled shRNA vector (Sc-shRNA) have been previously described^2, 3^. Cells were seeded in 10-cm plastic dishes and grown to 67% confluence at which point they were transfected with 4 μg of each vector using Lipofectamine2000 (Invitrogen). They were detached with trypsin after transfection for 48 h and seeded in the selection medium containing geneticin (G418) (500 μg/ml). Stable transfectants were selected at 4 weeks of culture.

**Cell viability assay**

The Cell Counting Kit-8 (CCK-8) (Dojindo Molecular Technologies, Inc. Beijing, China) was used to determine cell viability. Cells were seeded at 1 ×10^3^ cells /well in 96-well plates. At different time points, the culture medium was replaced with 100 μl of fresh medium containing 10μl of CCK-8 solution. The cells were further incubated for 2 h at 37°C, and the optical density (OD) at 450 nm was measured. The viability of cells was calculated by using a formula as below: (Experimental OD-Control OD)/Control OD × 100%. The experiment was repeated thrice.

**Cell fraction isolation**

The methods have been described previously^4^. Briefly, cells were rinsed with cold DEPC-treated PBS twice, then were scraped down and centrifuged to pellet the cells. Lysis buffer was added to the pellet and pipetted up and down ten times followed by being immediately centrifuged at 8,000 rpm for 1min. The supernatant was transferred to a new tube as the cytoplasm extracts, and the remainder was considered as nuclear fraction after washing once with wash buffer. The isolation was validated by Western blot analysis using an antibody against GAPDH for cytoplasm and antibody against laminA/C for nuclear extracts. The experiment was repeated thrice.

***In situ* hybridization for detecting miR-320a and TTN-AS1**

The *in situ* detection of miR-320a and TTN-AS1 expression was performed according to previously described methods with appropriate modifications^5, 6^. Double digoxigenin (DIG)-labelled locked nucleic acid probes for miR-320a (GAAGAACCGGGAAGAGAAG, RNA-Tm 85°C), TTN-AS1 (GGTCAGGGTGAT

AGGAAGTCCCAACAGTCCAGAAGGGCCACTGG, RNA-Tm 83°C), and a scrambled sequence (GTGTAACACGTCTATACGCCCA, RNA-Tm 87°C) as a negative control (Exiqon, Vedbaek, Denmark) were used. Cells were fixed with 4% paraformaldehyde and incubated with Proteinase-K (15 *μ*g/mL) at 37°C for 10 min. After being washed twice in phosphate buffered saline (PBS), cells were dehydrated in ethanol, blocked in prehybridization buffer (3% bovine serum albumin [BSA]) at 55°C for 30 min and incubated in hybridization buffer with probes (diluted at 1:2,000) at 55°C for 1 h. They were then washed with standard saline citrate buffer and blocked with 4% BSA for 1 h at room temperature. Positive signals were developed by overnight incubation with an anti-DIG Ab at 4°C, followed by incubations with fluorescein isothiocyanate (FITC) or Cy3-conjugated secondary antibodies. DAPI (4′,6-diamidino-2-phenylindole) was used to stain cell nuclei. Stained cells were visualized by laser scanning confocal microscopy. For detecting TTN-AS1 expression in tumor sections, similar methods were applied except for the use of a horseradish peroxidase (HRP)-conjugated secondary antibody, and immunoreaction with Sigma FAST DAB (3,3’-diaminobenzidine tetrahydrochloride) and CoCl_2_ enhancer tablets. All buffers in the above-described experiments were freshly prepared on the day of the experiment.

**RNA pulldown and** **RNA immunoprecipitation (RIP) assays**

RNA pulldown assay was carried out by using a Pierce™ Magnetic RNA-Protein Pull-Down Kit (Thermo Fisher Scientific, Shanghai, China). Briefly, RNAs were biotin-labeled and *in vitro* transcribed with Biotin RNA Labeling Mix and T7/SP6 RNA polymerase. Cells were lysed and incubated with biotinylated RNAs. Streptavidin agarose beads were added to each binding reaction, followed by a one-hour incubation at room temperature. Beads were washed and boiled in SDS (sodium dodecyl sulfate) buffer and retrieved proteins detected by Western blot analysis. RIP assay was conducted by using a Mana RIP RNA-Binding Protein Immunoprecipitation Kit (Millipore, Billerica, MA, USA). In brief, cells were lysed with RIP lysis buffer and incubated with RIP buffer, which contained magnetic beads with anti-Ago2 or anti-IgG Abs. IgG was used to be a negative control. After incubation for 2 h at 4°C, the precipitated RNAs were subjected to qRT-PCR for detecting the enrichment of TTN-AS1 and miR-320a.

**Transfection of oligonucleotides targeting miR-320a and TTN-AS1 expression vectors**

The double-stranded miR-320a mimics (5’- GCUUCGCUCCCCUCCGCCUUCUCU

UCCCGGUUCUUCCCGGAGUCGGGAAAAGCUGGGUUGAGAGGGCGAAAAAGGAUGAGGU-3’), specific miR-320a antagomiR (antagomiR-320a) (5’- AAAAGCUGGGUUGAGAGGGCGA-3’) and the negative control (NC) (5’-CAGUACUUUUGUGUAGUACAA-3’) oligonucleotides were purchased from GenePharma Co., Ltd., Shanghai, China). The full-length complementary DNA of human lncRNA-TTN-AS1 (Genbank no. NR_038272.1) was cloned into the lentiviral expression vector plenti-GIII-CMV-2A-Puro to generate TTN-AS1 expression vector and an empty lentiviral vector was used as bank control (Applied Biological Materials Inc., Richmond, BC). The short hairpin RNA (shRNA) for human TTN-AS-1 (sense5’-CACCGCGTTAGTCCACGGAGAATTGCGAACAATTCTCCGTGGACTAACGC-3’; anti-sense 5’-AAAGCGTTAGTCCACGGAGAATTGTTCGCAATTCT

CCGTGGACTAACGC-3’) was cloned into the lentiviral expression vector pGLV2/U6/puro, and a pGLV2/U6/puro vector with scrambled shRNA (sense 5’-ACCGCATCCATGTCCAAACTTACGCGAACGTAAGTTTGGACATGGATGC-3’; anti-sense 5’-AAAAGCATCCATGTCCAAACTTACGTTCGCGTAAGTTT

GGACATGGATGC-3’) was used as a negative control (GenePharma, Shanghai, China). Cells were grown to 60-70% confluence, and incubated with RNAs at a final concentration of 0.1 μM by using Lipofectamine^TM^ 2000 (Invitrogen) or 0.2 μg vectors using Effectene Transfection Reagent (QIAGEN) according to the manufacturer’s instructions, in serum-free media for 48 h and then subjected to assays.

**Assessment of cell cycle**

Cells were seeded at 5.0×10^5^ cells/well in six-well plates and underwent different treatments for 48 h, and then harvested and counted. A Cell Cycle kit (BD Biosciences, Beijing, China) was used to determine the percentages of cells at different phases of cell cycle by using flow cytometry with a Beckman Coulter Epics Altra II cytometer (Beckman Coulter, California, USA). The experiment was repeated thrice.

**Cell scratch assay**

Cells were seeded in 6-well culture plates. When the cells reached approximately 100% confluence, a scratch was made with a 10 μl sterile pipette tip, and the cells were cultured in serum-free RPMI-1640 medium for an additional 24 h. The images of the wound were recorded using a light microscope immediately after scratching and 18 h later. The horizontal migration abilities of the cells were quantified by measuring the wound widths of five different wound surfaces in each group using the Image-J analysis software. The experiment was performed three times.

**Transwell migration assay**

The migration of cells was determined using the Transwell migration assay. Transwell inserts (6.5 mm diameter and 8 μm pore size; Millipore) were loaded with 1 × 10^4^ cells in 200 μl of serum-free RPMI-1640 medium, and 600 μl of RPMI-1640 medium supplemented with 10% FBS was added to the lower chambers. The cells were allowed to migrate at 37 °C in a humidified CO_2_ incubator for 12 h. The cells remaining on the upper surface of the filter were then removed with cotton swabs, and the cells that migrated to the lower surface were stained with crystal violet (Beyotime Institute of Biotechnology, Haimen, China) for 20 min. Stained cells from five randomly chosen fields were counted under light microscopy.

**Quantitative reverse-transcription polymerase chain reaction (qRT-PCR)**

Methods have been described in detail previously^7, 8^. Briefly, total RNA was extracted from cell lysates or cytoplasm and nuclear extracts using TRIzol reagent (Invitrogen, Carlsbad, Calif., USA) according to the manufacturer’s protocols. RNA quantity and quality were measured using a NanoDrop ND-1000, and RNA integrity. RNA was reverse transcribed with oligo (dT) primer into cDNA with High Efficient Reverse Transcription Kit (Toyobo). The reaction mixtures for qRT-PCR were prepared with the primers as shown in Table S1. The PCR products were analyzed by MX3000P Real-time PCR systems (Stratagen, USA). Experiments were performed in triplicate, and data were calculated by ∆∆Ct methods.

**Luciferase assays and plasmid constructs**

The miR-320a targeting site in the enhancer/promoter regions of human lncRNA TTN-AS1gene was established by using RNAhybrid 2.2, a tool for finding minimum free energy hybridization (http://bibiserv.techfak.uni-bielefeld.de/rnahybrid) ^9^ (As shown in Fig. 6A). The TTN-AS1 fragment containing the putative miR-320a targeting sites was amplified by PCR using a pair of primers (5’- GCTAGCGCCACCATTATCAAGAGGGG-3’ and 5’- CTCGAGGTTGATTCCTTT

CCAGACCG-3’) and cloned into the NaeI and XhoI sites of pmirGLO Dual-Luciferase miRNA Target Expression Vector (Promega). This constructed reporter was named wild-type-TTN-AS1 (WT). To construct the mutant reporter plasmid, we used the Generate site-directed mutagenesis system (Invitrogen) to introduce mutations into the putative miR-320 targeting sites of the wild-type-TTN-AS1 vector (“CAAGAUUC AGCUUUA” was mutated to “GAAGCAAGGAGAGGG”). Luciferase reporter transfection and dual-luciferase assays were performed as described previously^7^. Briefly, the reporter vector plasmid was transfected into cells using Lipofectamine 2000. To correct transfection efficiency, an empty luciferase reporter vector without the miR-320a target was transfected in parallel. Luciferase activities in cells were measured by using a luciferase assay kit (Promega, Madison, WI) and were expressed as ratios of the luciferase activity of the reporter vector with miR-320a targeting sequence over the one without the miR-320a targeting sequence.

**Western blot analysis**

The methods have been described previously^3, 10^. Cells or tumor tissues were homogenized in protein lysate buffer (50 mM Tris pH 7.4, 100 μM EDTA, 0.25 M sucrose, 1%SDS, 1% NP40, 1μg/ml leupeptin, 1μg/ml pepstatin A and 100 μM phenyl methyl sulfonyl fluoride) and debris was removed by centrifugation at 10,000 × g for 10 min at 4^o^C. Protein concentrations of cell or tissue lysates were determined using the Bio-Rad protein assay (Bio-Rad, Richmond, CA, USA). Lysates were resolved on sodium dodecyl sulfate-polyacrylamide (SDS-PAGE) gels and electrophoretically transferred to polyvinylidene difluoride (PVDF) membranes. The membranes were blocked in TBST (137 mM NaCl, 20 mM Tris HCl [pH 7.6], and 0.1% [v/v] Tween 20) containing 5% (w/v) nonfat dry milk at 37^o^C for 2 h, and then incubated overnight with primary Abs, and subsequently with alkaline phosphatase-conjugated secondary Abs for 2 h at room temperature in the dark. They were developed with 5-bromo-4-chloro-3-indolyl phosphate (BCIP)/ nitro blue tetrazolium (NBT) (Tiangen Biotech Co. Ltd., Beijing, China). The density of each band was measured using a densitometric analysis program (FR200, Shanghai, China). In preliminary experiments, serial dilutions of lysates (containing 2.5, 5, 10, 20, 40 or 80µg protein) were immunoblotted; band intensities were measured and plotted against protein amounts to generate a standard curve, and the amount of protein for each blot was determined.

**Gelatin zymography assay**

The conditioned medium from an equal number of cells that had been incubated in serum-free medium for 48 h was collected and separated on 10% acrylamide gels containing 0.1% gelatin (Invitrogen). Gels were incubated in 2.5% Triton X-100 solution at room temperature with gentle agitation to remove SDS, and soaked in reaction buffer (50 mM Tris–HCl, pH 7.5, 150 mM NaCl, 10 mM CaCl_2_, and 0.5 mM ZnCl_2_) at 37°C overnight. After the reaction, gels were stained for 1 h with staining solution (0.1% Coomassie Brilliant Blue, 30% methanol, and 10% acetic acid) and then destained in the same solution without Coomassie Brilliant Blue. The gelatinolytic activity of MMP-2 and MMP-9 was visualized as a clear band against a dark background of stained gelatin.

**Animal experiments**

Six to 8-week-old male immunodeficient nude BALB/c mice (H-2b) were obtained from Vital River Laboratory Animal Technology (Beijing, China), and were housed at a specific pathogen-free facility in the Animal Research Center, the First Affiliated Hospital of Harbin Medical University, China. Two experiments were designed to examine the effects of TTN-AS1 knockdown and overexpression on the growth of RBE and FRN0201 tumors established in mice, respectively. RBE and FRN0201 cells were shown to express a high and low level of TTN-AS1, respectively. Cells (1 × 10^6^) were injected subcutaneously into the flanks of mice, and palpable tumors were monitored. Around 2-3 weeks later, mice bearing tumors with a volume of ~100 mm^3^ were randomly assigned to different groups (Each group had 8 mice). The TTN-AS1 knockdown study had 3 groups of animals, which received intratumoral injections of either control (scrambled shRNA expression vector + negative control oligonucleotides), shRNA-TTN-AS1 (shRNA-TTN-AS1 expression vector + negative control oligonucleotides) or shRNA-TTN-AS1 + antagomiR-320a; while the TTN-AS1 overexpression study comprised three groups of animals, which received intratumoral injections of either control (empty vector + negative control oligonucleotides), TTN-AS1 (TTN-AS1 expression vector ) or TTN-AS1 + miR-320a mimics. Each expression vector (100 μg) and one type of oligonucleotides (100μg) as above were mixed with 50 μl of Lipofectamine2000 to prepare the transfection solution, which was given on day 0, 5 and 10. Two mice from each group were sacrificed 2 days after gene delivery and tumors were harvested for detecting the expression of TTN-AS1 and miR-320a by *in situ* hybridization, and the expression of NRP-1 by immunohistochemistry. The remaining mice were further monitored for recording the size of tumors every 3 days and euthanized 15 days after treatments commenced.

**Immunohistochemistry of assessing gene expression in animal tumor tissues**

Cryosections (5 μm) of tumors harvested from animals were prepared, blocked with 3% BSA, and incubated with primary Abs at 4^o^C overnight. They were subsequently incubated for 30 min with appropriate secondary Abs using the Ultra-Sensitive TMS-P kit (Zhongshan Co., Beijing, China), and immunoreactivity developed with Sigma FAST DAB (3,3’-diaminobenzidine tetrahydrochloride) and CoCl_2_ enhancer tablets (Sigma-Aldrich, Shanghai, China). Sections were counterstained with hematoxylin, mounted, and examined by microscopy.

***In situ* Ki-67 proliferation index**

Tumor sections were immunostained with an anti-Ki-67Ab as above and examined to count Ki-67 positive cells in 10 randomly selected × 400 high-power fields under microscopy. The Ki-67 proliferation index was calculated according to the following formula: the number of Ki-67 positive cells/ the total cell count × 100%.

**Assessment of tumor vascularity**

Tumor sections were immunostained with an anti-CD31 Ab as above and examined under microscopy. Stained vessels were counted in ten blindly chosen random fields at 400 × magnification, and the microvessel density was recorded.

**Supplementary Tables**

**Table S1. Ten potential lncRNAs that are predicted to have binding sites with hsa-miR-320**

| **lncRNA** | **miRNA** | **Interaction** | **lncRNA Chr** | **Binding Start** | **Binding End** | **Energy (kCal/Mol)** | **Score** |
| --- | --- | --- | --- | --- | --- | --- | --- |
| lnc-TPPP2-1-2 | hsa-miR-320a | miRNA: 3' agcgggagaGUUGGGUCGAAAa 5'  \|\| \|:\|\|\|\|\|\|\|  lncRNA:5' atgagaagcCACCTCAGCTTTg 3' | chr14 | 21017166 | 21017187 | -15.54 | 153 |
| lnc-LRRC8C-2-1 | hsa-miR-320a | miRNA: 3' agcgGGAGAGUUGGGUCGAAAa 5'  :\|\| \| : \|\|\|\|\|\|\|  lncRNA:5' agaaTCTGCCCTTGCAGCTTTt 3' | chr1 | 89584656 | 89584677 | -19.59 | 142 |
| lnc-INADL-5-1 | hsa-miR-320a | miRNA: 3' agcgggAGAGUUG-GGUCGAAAa 5'  \|: :\|\|: \|\|\|\|\|\|\|\|  lncRNA:5' tcattgTTATAATGCCAGCTTTa 3' | chr1 | 61460606 | 61460628 | -14.98 | 151 |
| lnc-DPCD-9-2 | hsa-miR-320a | miRNA: 3' agCGGGAGAGUUGGGUCGAAAa 5'  \|\| \| \|\|\|\| \|\|\|\|\|\|\|  lncRNA:5' tgGCGCACTCA---CAGCTTTc 3' | chr10 | 101430431 | 101430449 | -17.48 | 152 |
| lnc-METTL23-1-1 | hsa-miR-320a | miRNA: 3' agcgggagagUUGG-GUCGAAAa 5'  :\|\|\| \|\|\|\|\|\|\|  lncRNA:5' tcaaacaggaGACCGCAGCTTTa 3' | chr17 | 76735858 | 76735880 | -13.57 | 147 |
| lnc-PI4KA-3-3 | hsa-miR-320a | miRNA: 3' agcGGGAG-AGUUGGGUCGAAAa 5'  \|\|: \| \|::\|\|\|\|\|\|\|\|\|  lncRNA:5' tcaCCTGCAGCGGCCCAGCTTTc 3' | chr22 | 20626122 | 20626100 | -25.20 | 158 |
| NF1P2 | hsa-miR-320a | miRNA: 3' agcgggagagUUGGGUCGAAAa 5'  \|\| \|\|\|\|\|\|\|  lncRNA:5' gattttgtgaAAAGCAGCTTTg 3' | chr15 | 21496898 | 21496877 | -10.43 | 144 |
| lnc-HDAC3-1-3 | hsa-miR-320a | miRNA: 3' agcgggaGAGUUGGGUCGAAAa 5'  \|\| ::\|\|\|\|\|\|\|\|  lncRNA:5' gttcaagCTGTGTCCAGCTTTt 3' | chr5 | 141640634 | 141640613 | -18.1 | 151 |
| TTN-AS1 | hsa-miR-320a | miRNA: 3' agcgggagagUUGGGUCGAAAa 5'  :\|::\|\|\|\|\|\|\|  lncRNA:5' caagtagcaaGATTCAGCTTTa 3' | chr2 | 178604837 | 178604858 | -13.1 | 148 |
| NEAT1 | hsa-miR-320a | miRNA: 3' agcgggagaguUGGGUCGAAAa 5'  ::\|\|\|\|\|\|\|\|  lncRNA:5' ccagaguggguGUCCAGCUUUg 3' | Chr11 | 65202217 | 65202238 | -18.38 | 152 |

Abbreviations: lncRNA, long non-coding RNA; lnc-TPPP2-1-1, tubulin polymerization promoting protein family member 2-1-1, lnc-LRRC8C-2-1, Leucine-rich repeat-containing protein 8C-2-1, lnc-INADL-5-1, InaD-like protein-5-1; lnc-DPCD-9-2, Primary ciliary dyskinesia D-9-2; lnc-METTL23-1-1, Methyltransferase-like protein 23-1-1; lnc-PI4KA-3-3, Phosphatidylinositol 4-kinase III alpha-3-3; NF1P2, neurofibromin 1 pseudogene 2; lnc-HDAC3-1-3, histone deacetylase 3; TTN-AS1; TTN Antisense RNA 1; NEAT1, nuclear paraspeckle assembly transcript 1.

**Table S2. Genes examined, primers used for qRT-PCR and PCR products in the study**

| Genes | Forward (5'→3') | Reverse (5'→3') | PCR product |
| --- | --- | --- | --- |
| lnc-TPPP2-1-2 | CCTTGCTTACGGTGGCCCAA | CTGACCAGTGCAGCATCCGT | 181 bp |
| lnc-LRRC8C-2-1 | TCCTGCACAGATTGGGGCAT | CAGTGCACCACAGCACACCT | 201 bp |
| lnc-INADL-5-1 | TCCTTACGGAGCCTGGCTGT | TTGTCCTTGCGGGCCATGTT | 267 bp |
| lnc-DPCD-9-2 | GGCCTCGGCGATTATGGACC | AAAGCTGTGAGTGCGCCAGT | 164 bp |
| lnc-METTL23-1-1 | ATATGCAAGGCCCGGGTGAC | TGCACTAGGCGCAGTTGTGT | 201 bp |
| lnc-PI4KA-3-3 | TTGCACAGTGATGCCAAG | GCAGGTAGGAGATGGGAAAG | 269 bp |
| NF1P2 | AGCAGTGACGGCAATGTGCT | TGTGCTCTGGAGGACCGAGG | 159 bp |
| lnc-HDAC3-1-3 | CATCAGCACAATGAAGGAGAC | TCTCATCTTCCCATCACCTAC | 215 bp |
| TTN-AS1 | GTCCACCAGCATTACAAGC | CCCCATTCCACATATTCACTC | 252 bp |
| NEAT1 | GACGAGATTAGATGGGCTCTTCTG | CGACCAAACACAGAAAAGACAACA | 137 bp |
| microRNA-320 | GCCGAGGCCTTCTCTTCCCGGTTC | CTCAACTGGTGTCGTGGAG | 67 bp |
| NRP-1 | GGAGCTACTGGGCTGTGAAG | ACCGTATGTCGGGAACTCTG | 208 bp |
| U6 | CTCGCTTCGGCAGCACA | AACGCTTCACGAATTTGCGT | 94 bp |
| U1 | GATACCATGATCACGAAGGTG | AAATTATGCAGTCGAGTTTCC | 111 bp |
| GAPDH | CACCCATGGCAAATTCCATGGCA | TCTAGACGGCAGGTCAGGTCCACC | 597 bp |

Explanations and abbreviations: Abbreviations: lncRNA, long non-coding RNA; lnc-TPPP2-1-1, tubulin polymerization promoting protein family member 2-1-1, lnc-LRRC8C-2-1, Leucine-rich repeat-containing protein 8C-2-1, lnc-INADL-5-1, InaD-like protein-5-1; lnc-DPCD-9-2, Primary ciliary dyskinesia D-9-2; lnc-METTL23-1-1, Methyltransferase-like protein 23-1-1; lnc-PI4KA-3-3, Phosphatidylinositol 4-kinase III alpha-3-3; NF1P2, neurofibromin 1 pseudogene 2; lnc-HDAC3-1-3, histone deacetylase 3; TTN-AS1, TTN Antisense RNA 1; NEAT1, nuclear paraspeckle assembly transcript 1; NRP-1, neuropilin-1; U6, U6 spliceosomal RNA; U1, U1 spliceosomal RNA; GAPDH, glyceraldehyde 3-phosphate dehydrogenase.

**References**

1. Kallioniemi OP, Wagner U, Kononen J, Sauter G. Tissue microarray technology for high-throughput molecular profiling of cancer. *Human molecular genetics* 2001, **10**(7)**:** 657-662.

2. Li L, Jiang X, Zhang Q, Dong X, Gao Y, He Y*, et al.* Neuropilin-1 is associated with clinicopathology of gastric cancer and contributes to cell proliferation and migration as multifunctional co-receptors. *Journal of experimental & clinical cancer research : CR* 2016, **35:** 16.

3. Wei Z, Jiang X, Qiao H, Zhai B, Zhang L, Zhang Q*, et al.* STAT3 interacts with Skp2/p27/p21 pathway to regulate the motility and invasion of gastric cancer cells. *Cellular signalling* 2013, **25**(4)**:** 931-938.

4. Wang P, Xu J, Wang Y, Cao X. An interferon-independent lncRNA promotes viral replication by modulating cellular metabolism. *Science (New York, NY)* 2017, **358**(6366)**:** 1051-1055.

5. de Planell-Saguer M, Rodicio MC, Mourelatos Z. Rapid in situ codetection of noncoding RNAs and proteins in cells and formalin-fixed paraffin-embedded tissue sections without protease treatment. *Nature protocols* 2010, **5**(6)**:** 1061-1073.

6. Li W, Dong X, He C, Tan G, Li Z, Zhai B*, et al.* LncRNA SNHG1 contributes to sorafenib resistance by activating the Akt pathway and is positively regulated by miR-21 in hepatocellular carcinoma cells. *Journal of experimental & clinical cancer research : CR* 2019, **38**(1)**:** 183.

7. He C, Dong X, Zhai B, Jiang X, Dong D, Li B*, et al.* MiR-21 mediates sorafenib resistance of hepatocellular carcinoma cells by inhibiting autophagy via the PTEN/Akt pathway. *Oncotarget* 2015, **6**(30)**:** 28867-28881.

8. Zhai B, Hu F, Jiang X, Xu J, Zhao D, Liu B*, et al.* Inhibition of Akt reverses the acquired resistance to sorafenib by switching protective autophagy to autophagic cell death in hepatocellular carcinoma. *Molecular cancer therapeutics* 2014, **13**(6)**:** 1589-1598.

9. Rehmsmeier M, Steffen P, Hochsmann M, Giegerich R. Fast and effective prediction of microRNA/target duplexes. *RNA (New York, NY)* 2004, **10**(10)**:** 1507-1517.

10. Li L, Jiang X, Zhang Q, Dong X, Gao Y, He Y*, et al.* Neuropilin-1 is associated with clinicopathology of gastric cancer and contributes to cell proliferation and migration as multifunctional co-receptors. *Journal of experimental & clinical cancer research : CR* 2016, **35**(1)**:** 16.
