# Supplementary material for: LncRNA TTN-AS1 promotes the progression of cholangiocarcinoma via the miR-320a/neuropilin-1 axis
Source: Cell Death Dis. 2020 Aug 15;11(8):637. doi: 10.1038/s41419-020-02896-x (PMC7429853; doi:10.1038/s41419-020-02896-x)
Supplement: Supplementary file 2 — Supplementary Figure Legends [file 41419_2020_2896_MOESM2_ESM.docx]

**Supplementary Figure Legends**

**Fig. S1 The expression of NRP-1 in clinical CCA and adjacent normal tissues examined by tissue microarray and immunohistochemistry.** (A) One core region (2 mm × 2 mm) from each of 39 pairs of paraffin-embedded CCA and normal tissue samples was perforated to prepare the tissue microarrays, which were subjected to immunohistochemistry. (B) Representative images were from CCA and corresponding adjacent normal tissues were immunostained with an anti-NRP-1 Ab. Scale bar, 200 μm.

**Fig. S2 The expression of NRP-1 and miR-320 in CCA cells and the correlation.** (A) A panel of human CCA cells and normal bile duct epithelial HINEC cells were subjected to immunoblotting to detect NRP-1 expression, and band density was normalized to β-actin. (B, C) The above cells were subjected to qRT-PCR for measuring the expression of NRP-1 mRNA (B) and miR-320 (C). (D) The correlation between miR-320 and NRP-1 mRNA expression was analyzed by using a Pearson test and plotted.

**Fig. S3 TTN-AS1 is overexpressed in CCA cells and correlates with miR-320 and NRP-1.** (A) RBE and HIBEC cells were subjected to qRT-PCR for detecting the expression of ten potential lncRNAs. (B) The fold changes of each lncRNA between RBE and HIBEC cells are plotted. (C) The expression levels of TTN-AS1 in a panel of CCA cells and normal bile duct epithelial HINEC cells were measured. (D, E) The correlation of TTN-AS1 with miR-320 (D) and NRP-1 mRNA (E) was analyzed by using a Pearson test and plotted. “*” (P<0.05) and “**” (P<0.001) indicate a significant difference.

**Fig. S4 The expression of TTN-AS1 and miR-320 in CCA cells transfected with miR-320 mimics or TTN-AS1 shRNA.** (A) RBE and HCCC-9810 transfected with negative control (NC) or miR-320 mimics were subjected to qRT-PCR for measuring the expression of TTN-AS1. (B) RBE and HCCC-9810 transfected with NC or shRNA-TTN-AS1 were subjected to qRT-PCR for measuring the expression of miR-320a. “**” (P<0.001) indicates a significant difference from the negative control.

**Fig. S5 TTN-AS1 interacts with miR-320a in CCA cells.** (A) The diagram of dual-luciferase reporter vectors containing wild-type (AT) or mutant (MU) lncRNA TTA-AS1 with miR-320 binding site, and the predicted paring of Hsa-miR-320a and lncRNA TTN-AS1. (B) Relative luciferase activities in RBE cells co-transfected with miR-320a mimics or negative control (NC) and luciferase reporters containing TTN-AS1-WT or TTN-AS1-MU. (C) The predicted paring of wild-type miR-320a (miR-320a-WT) and mutant miR-320a (miR-320a-MU) with TTN-AS1. (D, E) RNA pull-down assay using biotin-labeled TTN-AS1 or biotin-labeled miR-320a in RBE cells. (D) Fold enrichment of miR-320a in RBE cells transfected with NC, TTN-AS1-WT, or TTN-AS1-MU in RBE cells. (E) Relative expression of TTN-AS1 in RBE cells transfected with NC, miR-320a-WT, or miR-320a-MU. “**” (P<0.001) indicates a significant difference from corresponding negative controls.

**Fig. S6 TTN-AS1 negatively regulates the expression of miR-320a in a one-way manner.** RBE cells were transfected with either TTN-AS1 expression vector, shRNA-TTN-AS1, miR-320a mimics, antagomiR-320a, or various combinations for 48 h. Empty vectors and negative control oligonucleotides served as controls. Cells were subjected to qRT-PCR and the expression levels of TTN-AS1 and miR-320a were normalized to mock-treated cells. The comparisons were made with a one-way ANOVA followed by a Tukey post-hoc test. “**” (P<0.001) indicates a significant difference. “NS” refers to “no significant difference.

**Fig. S7 MiR-320a inhibits the proliferation of CCA cells by downregulating NRP-1.** (A, B) RBE, RBE-Sc and RBE-NRP^low^ cells were subjected to fluorescence immunocytochemistry for visualizing (A) and immunoblotting (B) for detecting NRP-1 expression. Scale bar, 50 μm. (C) The above cells were incubated for 7 days, and their viability was measured daily. (D, E) RBE cells were mock-transfected or transfected with negative control (NC) oligonucleotides or miR-320 mimics for 48 hours. The expression of NRP-1 was detected by immunoblotting (D), and cell viability was measured and normalized to mock-treated controls (E). Immunoblotting band density was normalized to β-actin. The comparisons were made with a one-way ANOVA followed by a Tukey post-hoc test. “**” (P<0.001) indicates a significant difference.

**Fig. S8 TTN-AS1 promotes cell proliferation via miR-320a/NRP-1.** (A, B) RBE and HCCC-9810 cells were transfected with scrambled shRNA (control), shRNA-TTN-AS1, or shRNA-TTN-AS1 + antagomiR-320a for 48 hours. (C, D) FRN0201 cells were transfected with the empty lentiviral vector (control), TTN-AS1 expression vector or TTN-AS1 expression vector + miR-320a mimics for 48 hours. (A, C) Cells were subjected to CCK-8 assays and cell viability was normalized to mock-treated cells. (B, D) Cells were subjected to immunoblotting, and the density of each band was normalized to β-actin. “**” (P<0.001) vs. controls, “#” (P<0.05) and “##” (P<0.001) vs. shRNA-TTN-AS1, and “φφ” (P<0.001) vs. TTN-AS1.

**Fig. S9 TTN-AS1 promotes cell cycle progression at the G1/0 phase.** Mock-treated RBE cells (A) and RBE cells transfected with scrambled shRNA (control) (B), shRNA-TTN-AS1 (C) or shRNA-TTN-AS1 + antagomiR-320a (D) were subjected to flow cytometry for detecting cell cycle distribution (A-D) and percentages of cells at different phases were plotted (E). “**” (P<0.001) vs. controls, and “##” (P<0.001) vs. shRNA-TTN-AS1.

**Fig. S10 TTN-AS1 promotes cell migration.** FRN0201 cells were transfected with the empty lentiviral vector (control), TTN-AS1 expression vector or TTN-AS1 expression vector + miR-320a mimics for 48 hours. Mock-treated cells served as controls as well. (A) Cells were subjected to Transwell migration (upper panel, scale bar, 100 μm) and scratch (lower panel, scale bar, 100 μm) assays. (B) Numbers of migrating cells were counted. (C) Scratch gaps were quantified. “**” (P<0.001) vs. controls, and “φ” (P<0.05) and “φφ” (P<0.001) vs. TTN-AS1.

**Fig. S11 TTN-AS1 plays a promoting role in the growth and angiogenesis of CCA tumors.** Subcutaneous CCA tumors were established in mice by inoculation of FRN0201 cells and received respective treatments as described in Materials and Methods. (A) The growth curve of FRN0201 tumors was recorded. (B-E) Tumors were resected at the end of experiments. (B) The weight of tumors was recorded. (C) Two mice were sacrificed from each group to harvest tumors 2 days after treatments and the expression of TTN-AS1 and miR-320a was examined by *in situ* hybridization (Magnification × 200; Scale bar, 100 μm), and NRP-1 expression by immunohistochemistry (Magnification × 400; Scale bar, 50 μm). (D) Illustrated are representative tumor sections immunostained by Abs against Ki-67 and CD31, respectively. Magnification × 400. *In situ* cell proliferation index (E) and tumoral microvessel density (F) were quantified. “n”, number of mice. Scale bar, 100 μm. “**” (P<0.001) vs. controls; “φ” (P<0.05) and “φφ”(P<0.001) vs. TTN-AS1.
