# Supplementary figures and images for: LncRNA TTN-AS1 promotes the progression of cholangiocarcinoma via the miR-320a/neuropilin-1 axis
Source: Cell Death Dis. 2020 Aug 15;11(8):637. doi: 10.1038/s41419-020-02896-x (PMC7429853; doi:10.1038/s41419-020-02896-x)

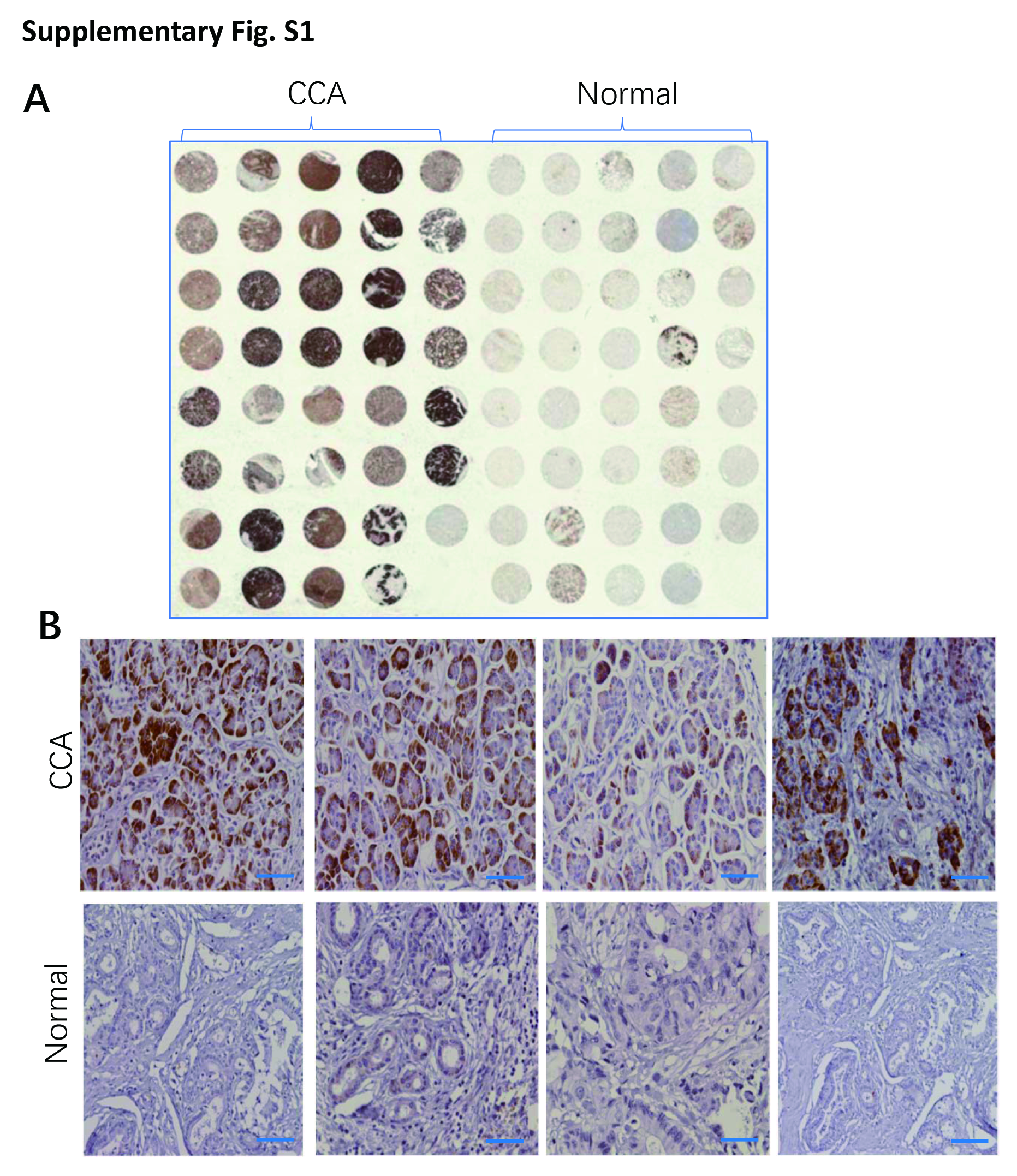

Supplement: Supplementary file 3 — Supplementary Fig. S1 [file 41419_2020_2896_MOESM3_ESM.tif]

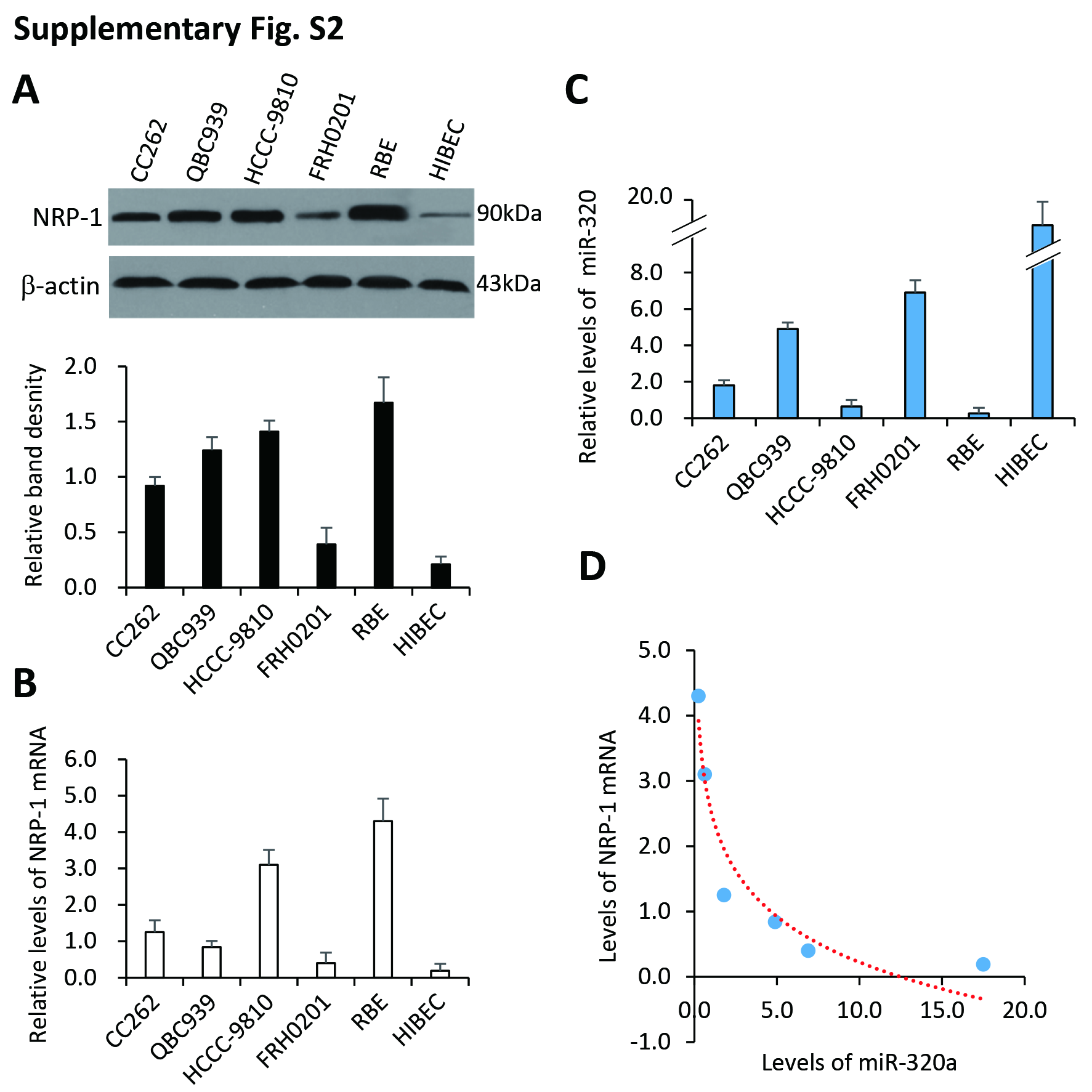

Supplement: Supplementary file 4 — Supplementary Fig. S2 [file 41419_2020_2896_MOESM4_ESM.tif]

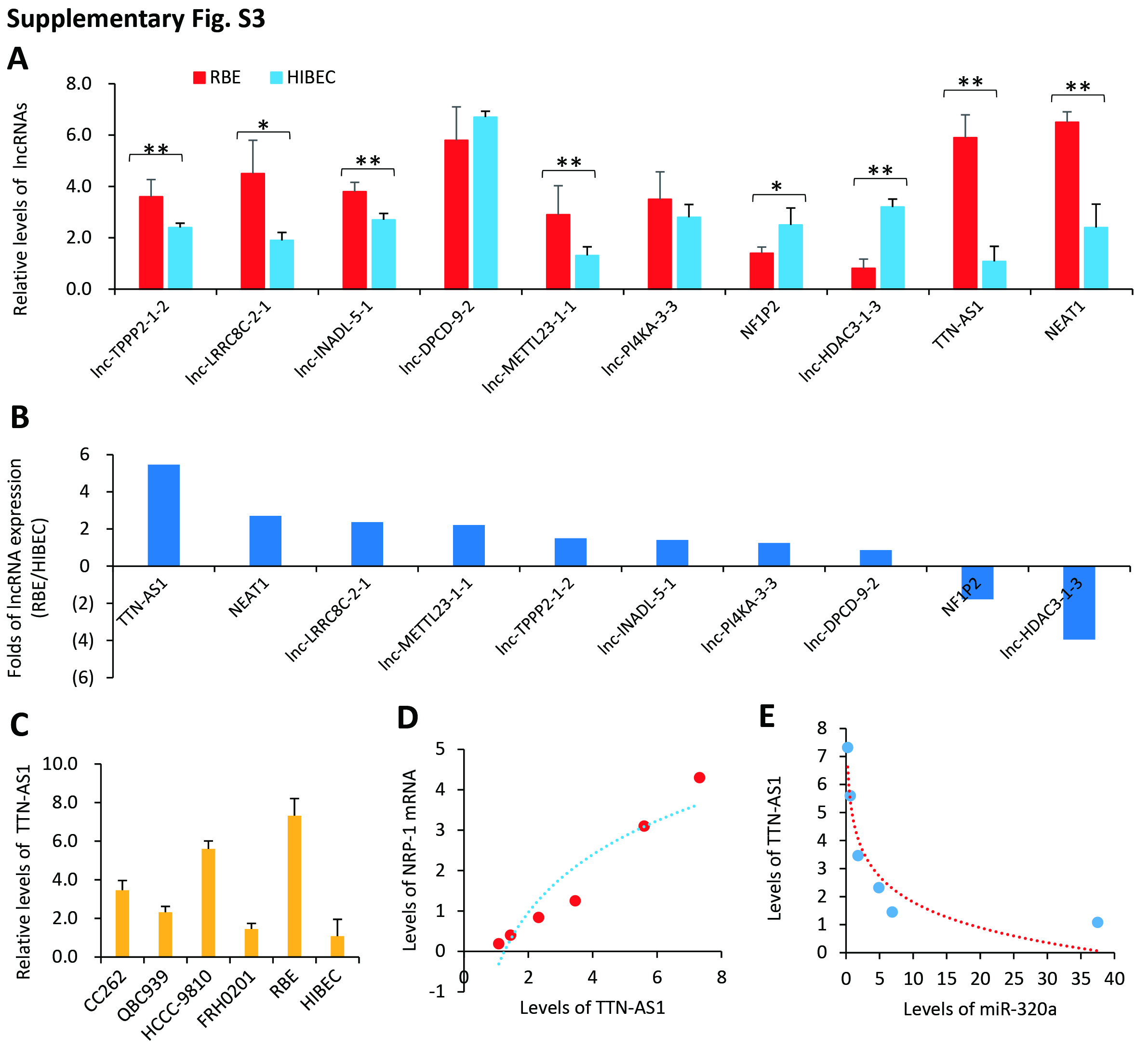

Supplement: Supplementary file 5 — Supplementary Fig. S3 [file 41419_2020_2896_MOESM5_ESM.tif]

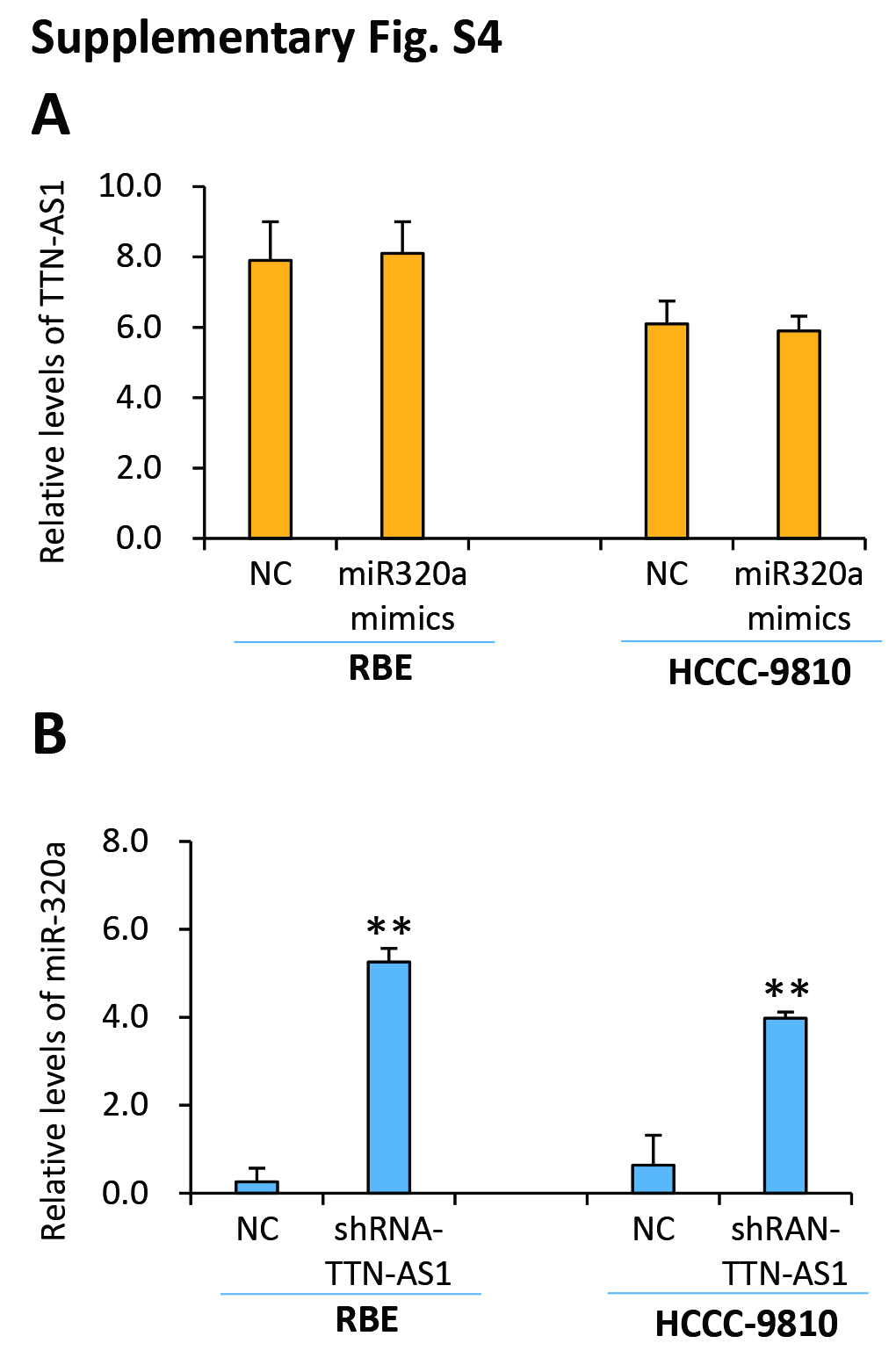

Supplement: Supplementary file 6 — Supplementary Fig. S4 [file 41419_2020_2896_MOESM6_ESM.tif]

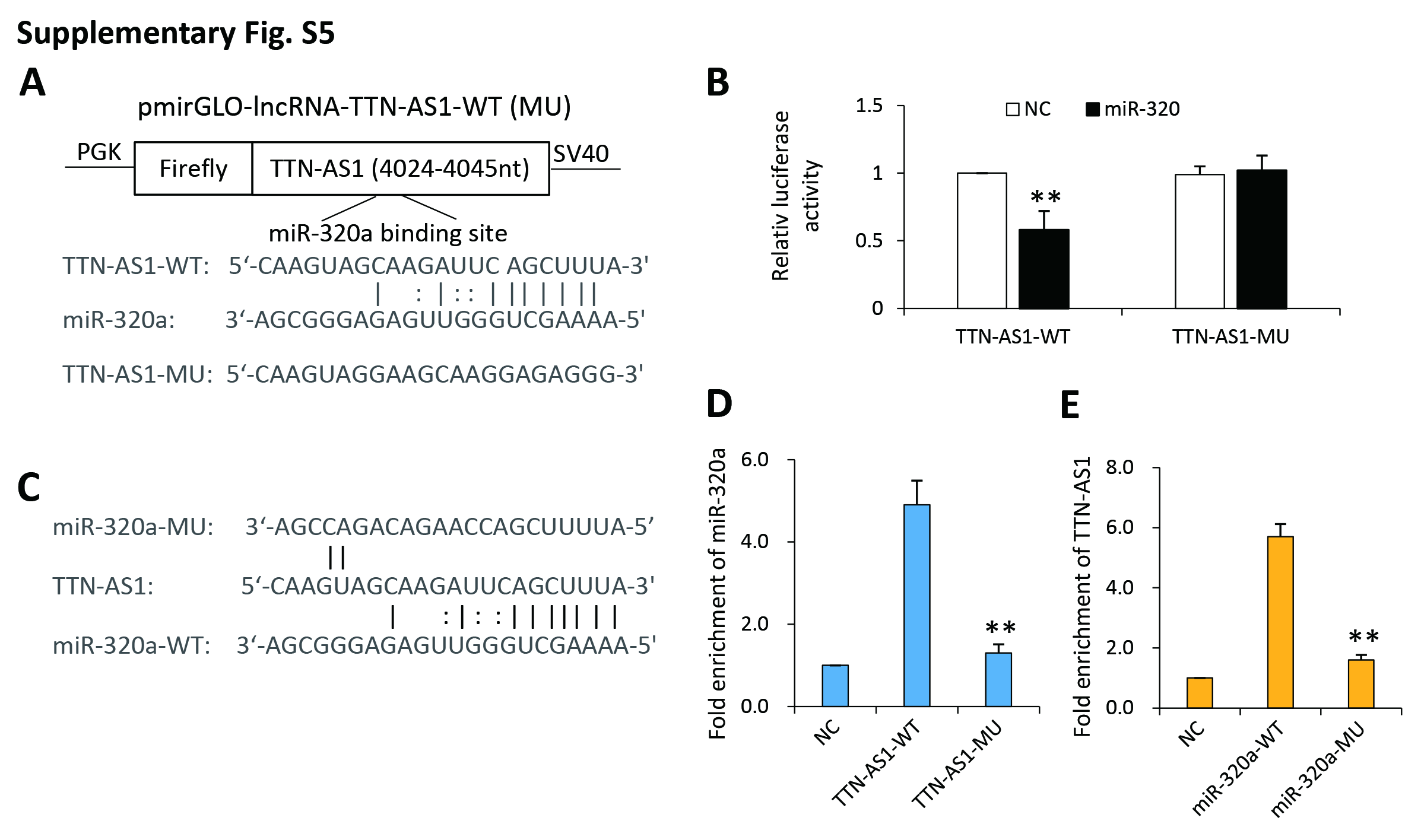

Supplement: Supplementary file 7 — Supplementary Fig. S5 [file 41419_2020_2896_MOESM7_ESM.tif]

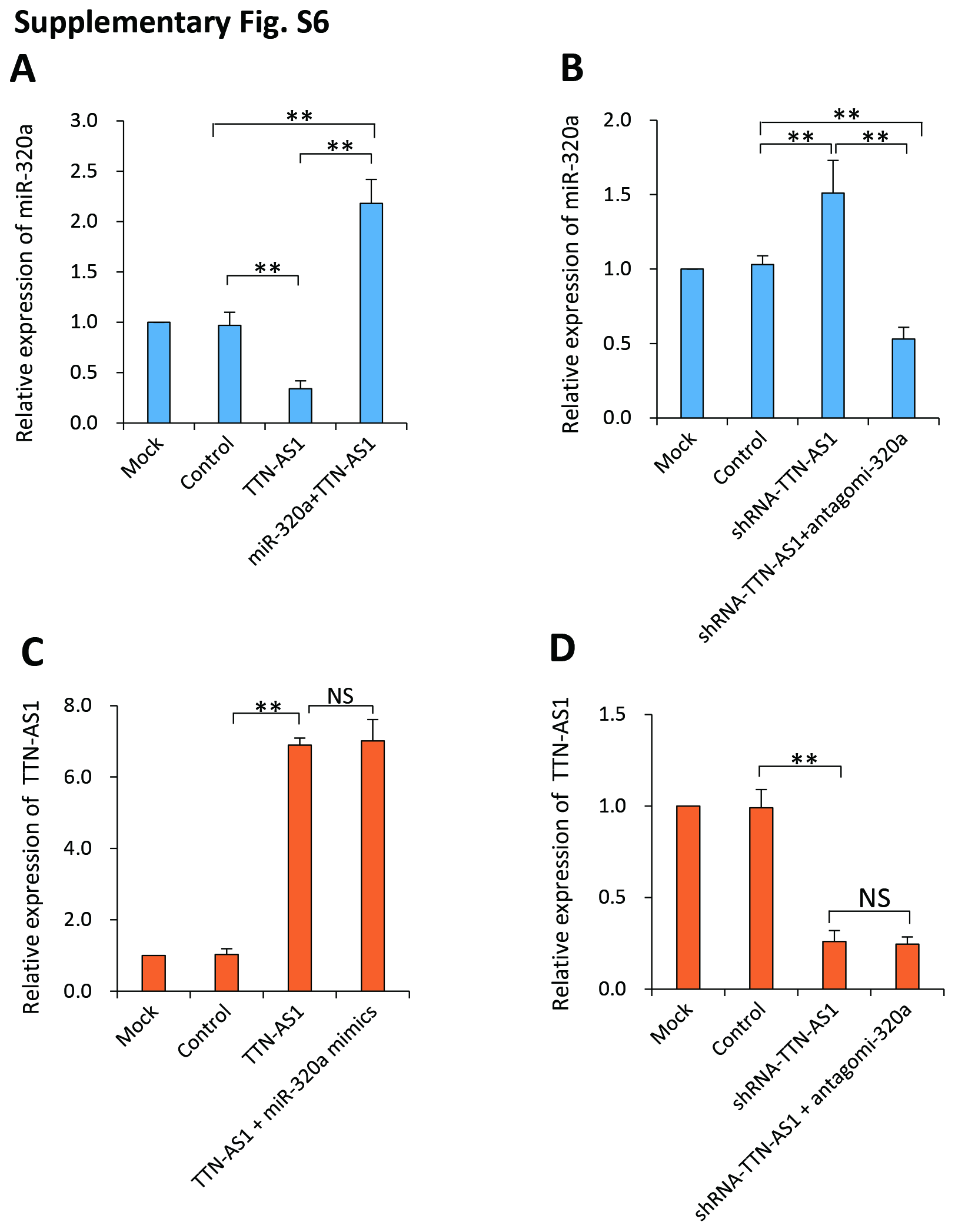

Supplement: Supplementary file 8 — Supplementary Fig. S6 [file 41419_2020_2896_MOESM8_ESM.tif]

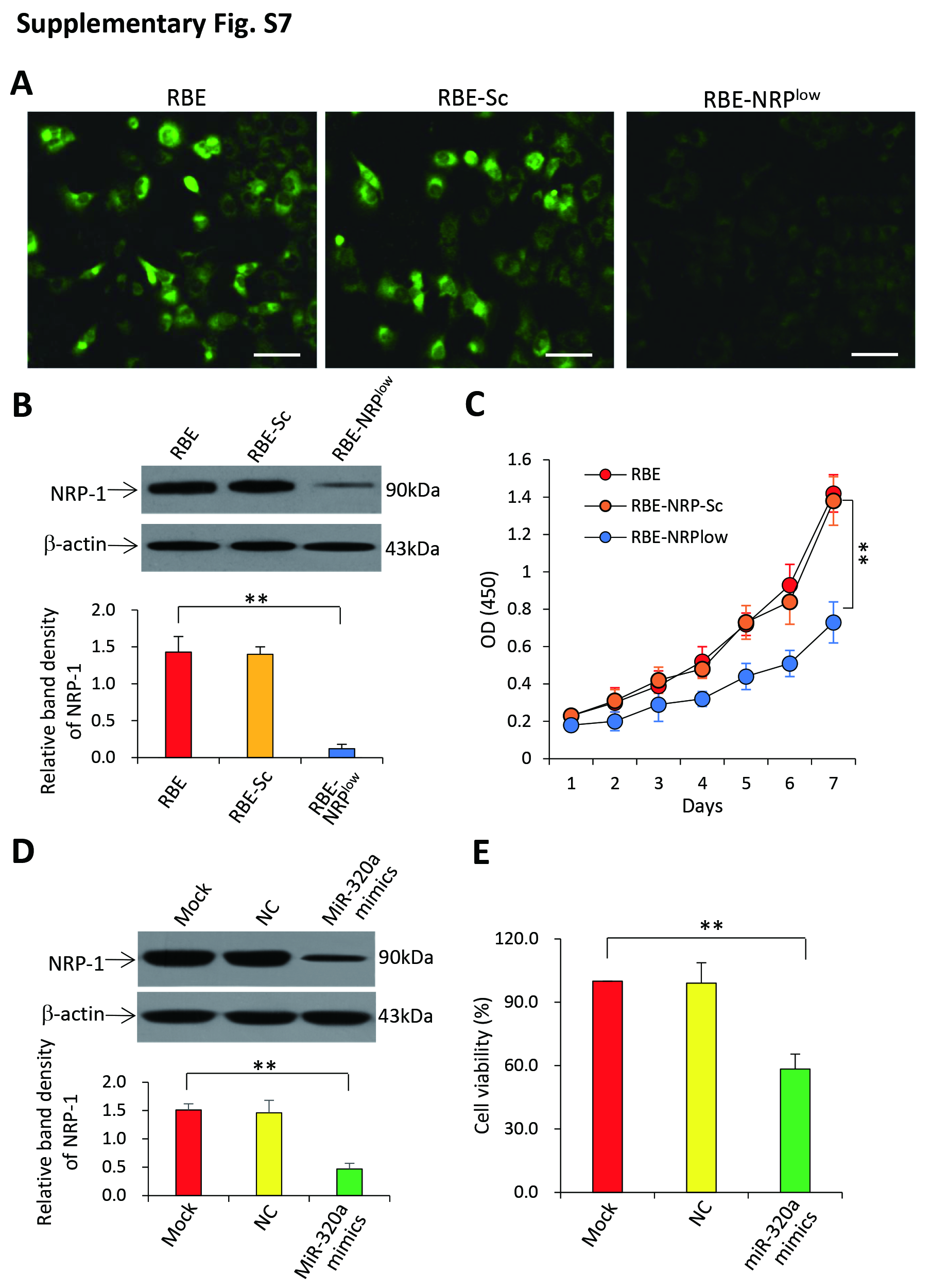

Supplement: Supplementary file 9 — Supplementary Fig. S7 [file 41419_2020_2896_MOESM9_ESM.tif]

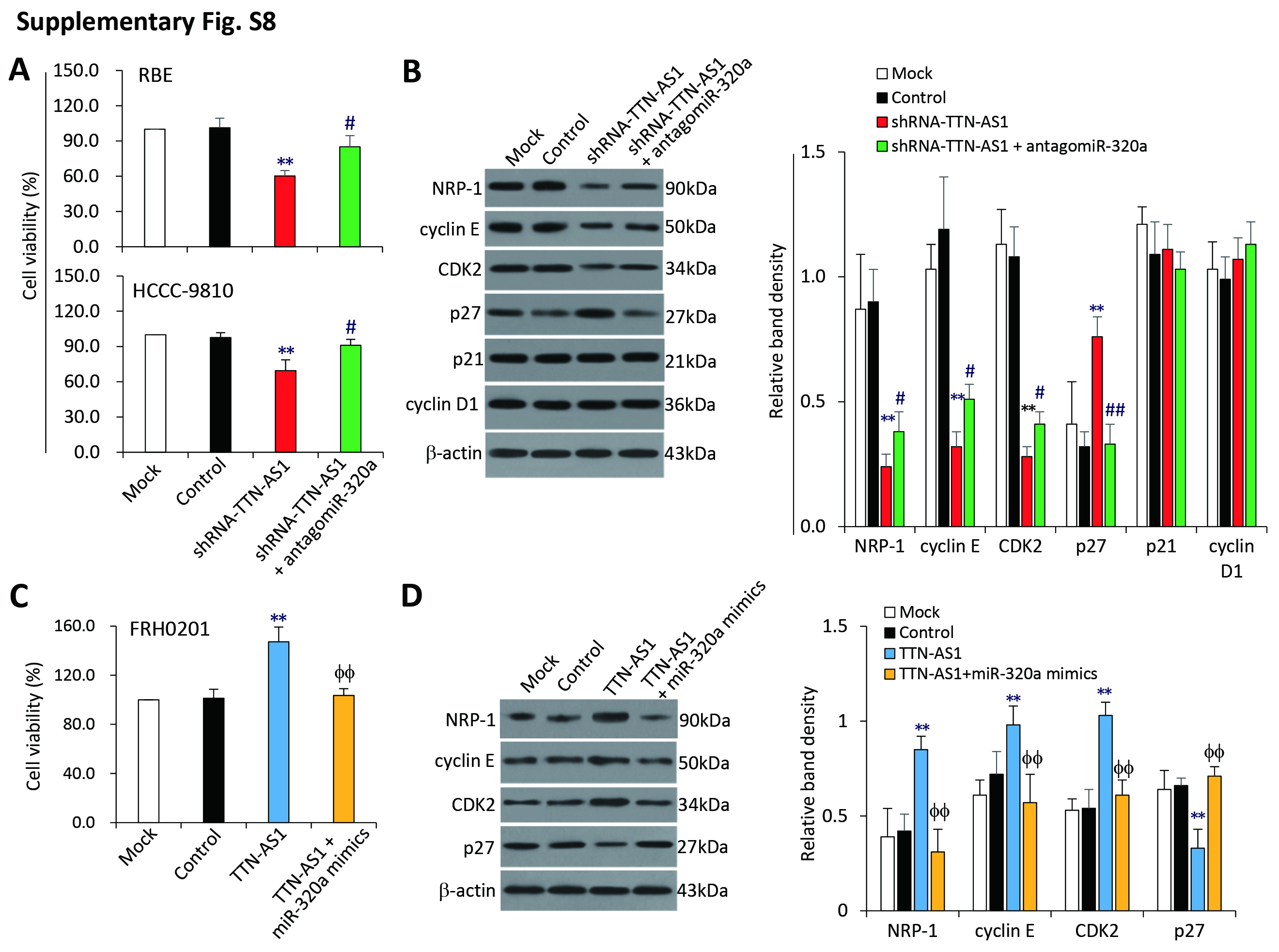

Supplement: Supplementary file 10 — Supplementary Fig. S8 [file 41419_2020_2896_MOESM10_ESM.tif]

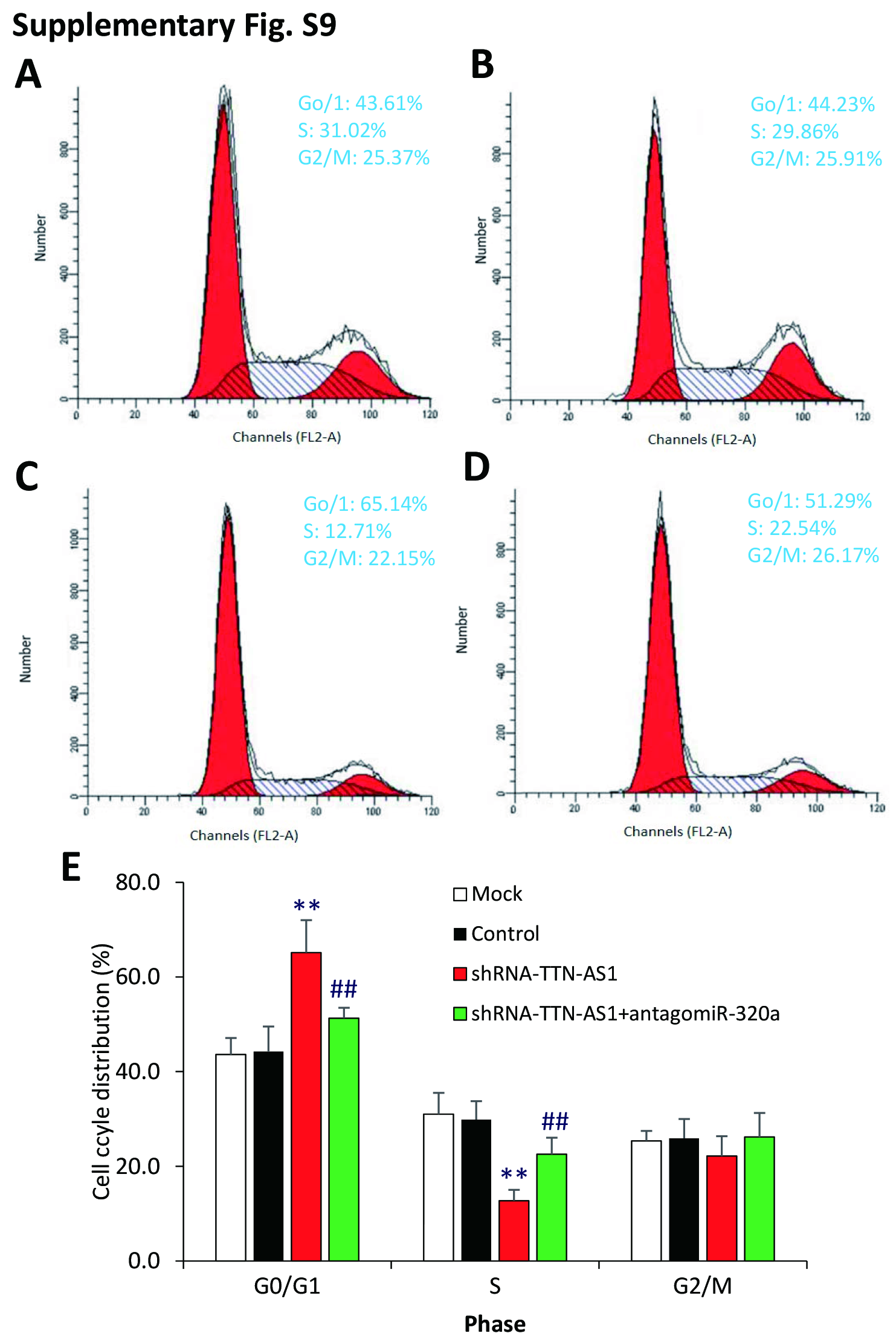

Supplement: Supplementary file 11 — Supplementary Fig. S9 [file 41419_2020_2896_MOESM11_ESM.tif]

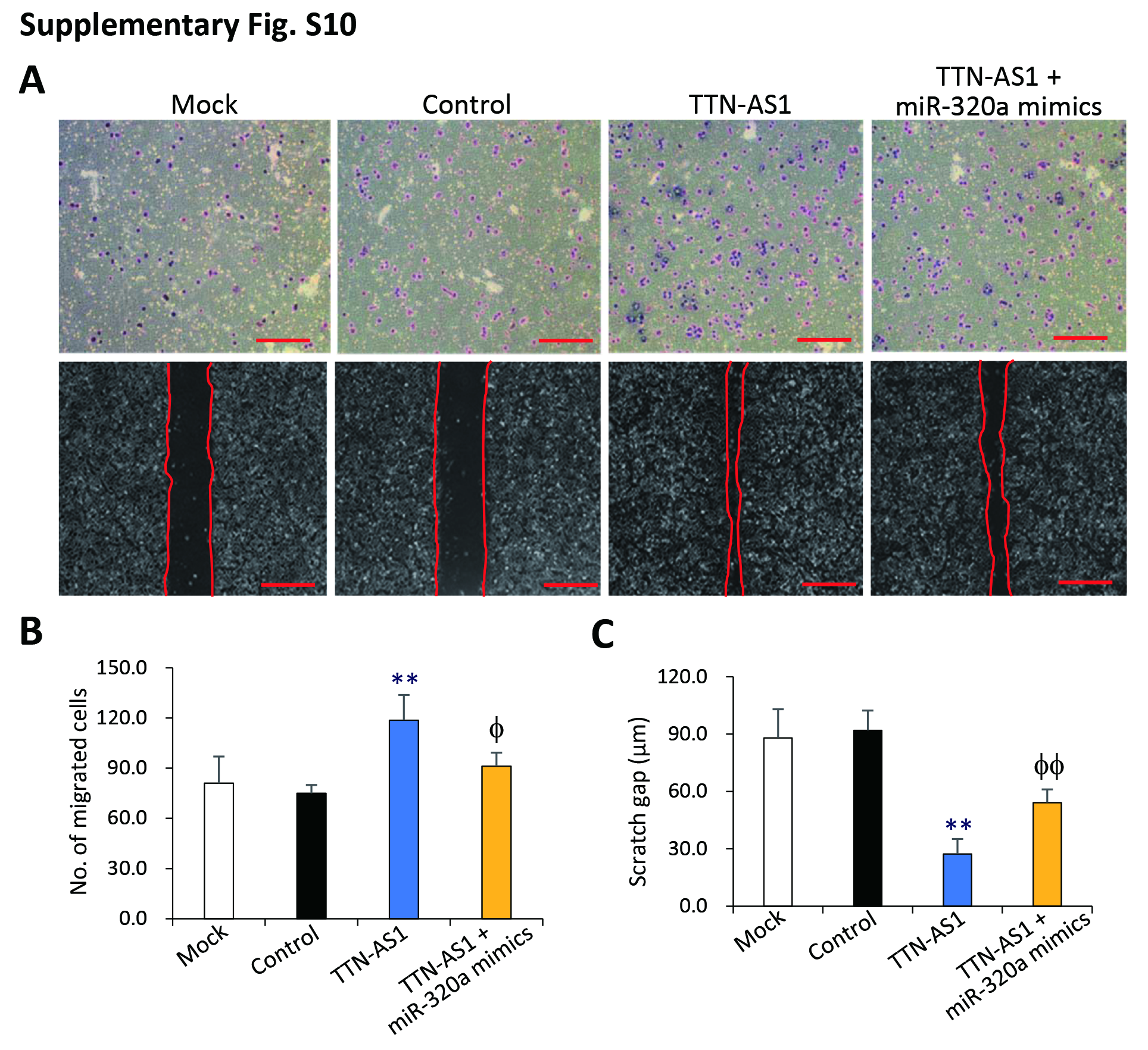

Supplement: Supplementary file 12 — Supplementary Fig. S10 [file 41419_2020_2896_MOESM12_ESM.tif]

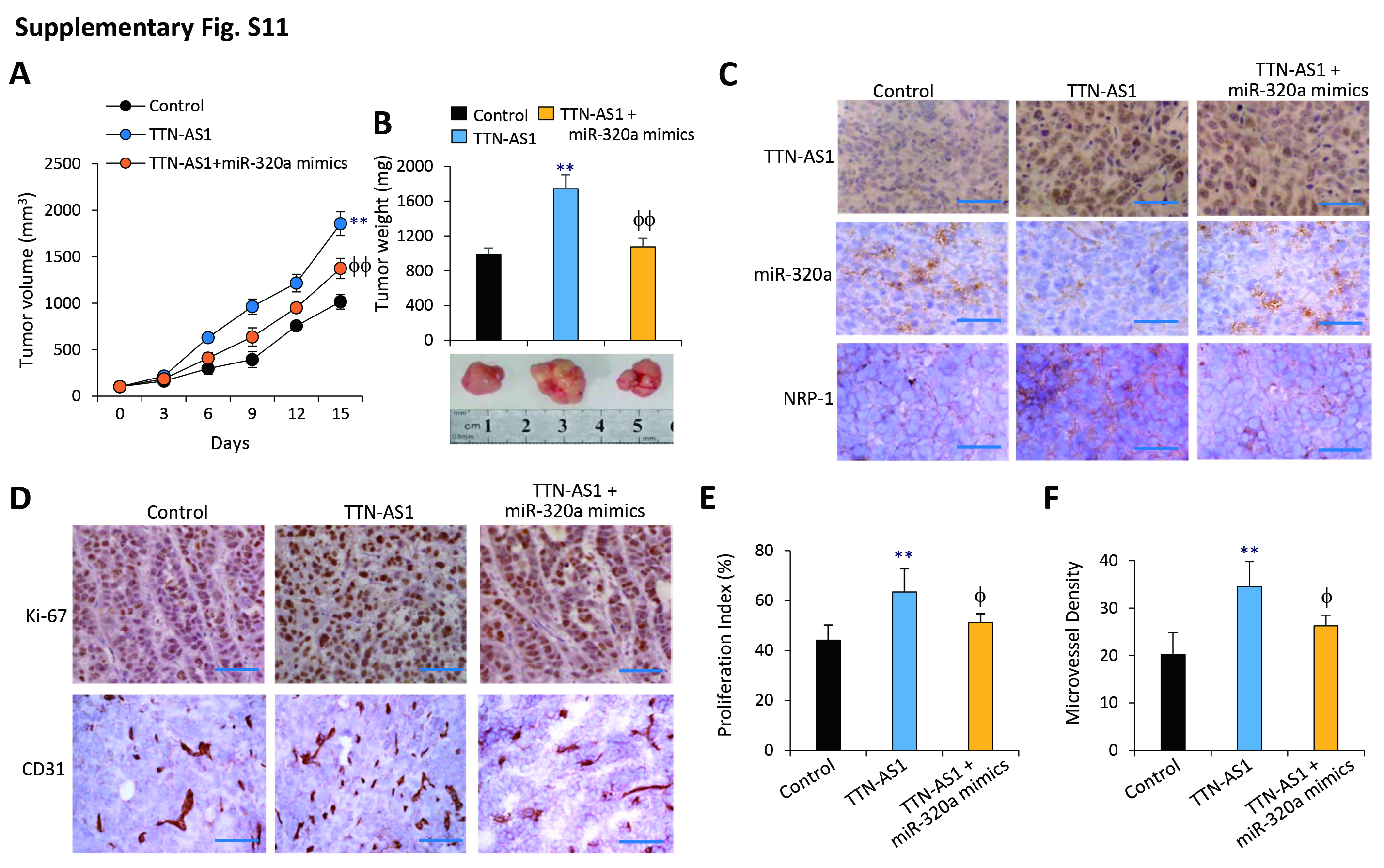

Supplement: Supplementary file 13 — Supplementary Fig. S11 [file 41419_2020_2896_MOESM13_ESM.tif]
